# Integrated analysis of transcriptome and proteome reveal that PDCoV infection induces autophagy-dependent ferroptosis to facilitate viral replication

**DOI:** 10.1186/s13567-026-01724-y

**Published:** 2026-05-18

**Authors:** Xiaozhu Yang, Xingyu Mi, Wei Liu, Farwa Zainab, Minrui Wu, Hanwei Yin, Mengyuan Liu, Ting Zhang, Zilong Sun, Ding Zhang, Pan Tang, Tao Song, Liqiang Duan, Yibo Xi, Chenyang Wang, Wei Li, Haidong Wang, Bo Yang

**Affiliations:** 1 Shanxi Key Laboratory of Animal Disease Research, Prevention and Control, College of Veterinary Medicine, Shanxi Agricultural University, Taigu, Jinzhong, 030801 China; 2 GloriousMed Clinical Laboratory Co. Ltd, Shanghai, 200120 China; 3 College of Veterinary Medicine, Shanxi Agricultural University, Taigu, Jinzhong, 030801 Shanxi China; 4 Shanxi Key Lab for Modernization of TCVM, College of Veterinary Medicine, Shanxi Agricultural University, Taigu, Jinzhong, 030801 Shanxi China; 5 Institute of Animal Husbandry and Veterinary Science, Shanghai Academy of Agricultural Sciences, Shanghai, 201106 China; 6 College of Animal Science and Technology, Hebei Normal University of Science and Technology, Qinhuangdao, 066600 China; 7 Shanxi Academy of Advanced Research and Innovation, Taiyuan, 030000 China; 8 School of Management, Shanxi Medical University, Taiyuan, 030000 China

**Keywords:** PDCoV, transcriptomic analysis, proteomic analysis, autophagy, ferroptosis

## Abstract

Porcine deltacoronavirus (PDCoV) is an emerging enteropathogenic coronavirus that causes severe diarrhea in swine industries worldwide. However, the interactions between PDCoV and host cells remain poorly understood. In this study, we employed transcriptomic and proteomic analyses to investigate host responses to PDCoV infection. Our results identified 1448 differentially expressed genes (DEGs) at 1.5 h post-PDCoV infection and 11,753 DEGs, along with 898 differentially expressed proteins (DEPs) at 18 h post-PDCoV infection. Furthermore, several signaling pathways, including innate immunity, autophagy, and ferroptosis, were primarily enriched following an integrated analysis of the transcriptome and proteome. Protein–protein interaction (PPI) analysis indicated that proteins closely associated with these pathways, such as interferon-induced protein with tetratricopeptide repeats 1 (IFIT1), myxovirus resistance 2 (MX2), interferon-stimulated gene 15 (ISG15), radical *S*-adenosyl methionine domain containing 2 (RSAD2), 2′-5′-oligoadenylate synthetase like (OASL), autophagy related 14 (ATG14), and glutathione peroxidase 4 (GPX4), were central to the interaction network. Importantly, we demonstrated that autophagy and ferroptosis were induced upon PDCoV infection, and that inhibition of autophagy significantly suppressed the induction of PDCoV-induced ferroptosis, which decreases the viral proliferation. Overall, our findings provide a comprehensive overview of transcriptomic and proteomic changes following PDCoV infection and enhance the understanding of PDCoV pathogenesis, which will be beneficial for improving strategies for the prevention and control of PDCoV infection.

**Supplementary Information:**

The online version contains supplementary material available at 10.1186/s13567-026-01724-y.

## Introduction

Porcine deltacoronavirus (PDCoV) belongs to the genus *Deltacoronavirus* in the family *Coronaviridae* and is recognized as a significant intestinal and respiratory pathogen [1]. Since the initial outbreak of PDCoV was announced in the USA, this virus has spread to many countries worldwide [2–5]. Similar to other swine enteric coronaviruses, such as porcine epidemic diarrhea virus (PEDV) and porcine transmissible gastroenteritis virus (TGEV), PDCoV cause vomiting, watery diarrhea, dehydration, and high mortality rates in piglets, leading to considerable economic losses for the global swine industry [6]. Symptomatic PDCoV infection has been observed in calves, mice, and chickens [7, 8]. In 2021, PDCoV was detected in Haitian children with acute undifferentiated febrile illness, suggesting that PDCoV possesses zoonotic potential and raising public health concerns [9, 10].

An in-depth understanding of virus–host interactions provides a theoretical foundation for the control of viral infections. To date, studies on the interactions between PDCoV and the hosts have been reported [11]. It was reported that the PDCoV N protein directly targets RIG-I to interfere with its binding to dsRNA, thus inhibiting the production of interferon (IFN)-β [12]. PDCoV NS7a interacts with inhibitor of nuclear factor kappa-B kinase subunit epsilon (IKKε), significantly disrupting the interaction between IKKε and interferon regulatory factor 3 (IRF3) [13]. These studies elucidated the mechanisms by which PDCoV suppresses the innate immune response within host cells to promote its replication. Moreover, PDCoV infection also induces apoptosis and autophagy to regulate self-replication [14, 15]. However, much remains to be understood regarding the interactions between PDCoV and hosts.

In recent years, integrated analysis of multi-omics data has provided a comprehensive view of virus-host interactions and facilitated the identification of potential biomarkers for controlling viral infections [16, 17]. For instance, combined transcriptomic and metabolomic analysis showed that multiple signaling pathways are involved in PDCoV infection, such as mitogen-activated protein kinase (MAPK), the phosphoinositide 3-kinase (PI3K/Serine/Threonine Kinase) (AKT) signaling pathway, and ferroptosis [18]. Comparative transcriptomic and proteomic analyses proved that IFN-λ1 is a more potent inducer of ISGs than IFN-α against PEDV infection [19]. These studies confirmed the significant role of integrated transcriptomic and proteomic analyses in uncovering potential antiviral targets.

Autophagy and ferroptosis are two critical cellular processes that influence cell survival, death, and stress responses [20–22]. Autophagy is a conserved lysosomal degradation pathway that is essential for maintaining cellular homeostasis, while ferroptosis is an iron-dependent form of regulated cell death characterized by lipid peroxidation [23, 24]. Numerous studies have shown that viral replication is closely related to autophagy or ferroptosis. For example, an antiviral mechanism has been identified in which the host B-cell translocation gene 3 (BTG3) targets the viral S2 protein to inhibit the proliferation of PEDV through the autophagy signaling pathway [25]. Ras-selective lethal 3 (RSL3) inhibits PEDV replication by activating ferroptosis [26]. As for PDCoV, previous studies have reported that PDCoV induced autophagy to facilitate viral replication via the PI3K/Akt/mechanistic target of rapamycin (mTOR) and p38 signaling pathway [27, 28]. However, the inter-regulation of autophagy and ferroptosis and its impact on viral replication during PDCoV infection remain unexplored.

In the present study, differentially expressed genes (DEGs) and differentially expressed proteins (DEPs) were screened in PDCoV-infected LLC-PK1 cells at 1.5 and 18 h by transcriptomic and proteomic analysis. Further analysis by integration analysis of transcriptome and proteome showed that innate immunity, autophagy, and ferroptosis signaling pathways were enriched. Additionally, we demonstrated that PDCoV infection significantly activated autophagy and ferroptosis, and that inhibition of autophagy suppressed PDCoV-induced ferroptosis, which further reduced viral replication. These findings provide new insights to understand the interactions between PDCoV and hosts.

## Materials and methods

### Cells, virus, and drugs

LLC-PK1 cells were obtained from the American Type Culture Collection (ATCC) and were cultured in Dulbecco’s modified Eagle medium (DMEM; Gibco, USA) supplemented with 1% antibiotic–antimycotic (Gibco, USA) and 10% fetal bovine serum (FBS; Gibco, Australia). The PDCoV strain (CHN/SX-Y/2023, GenBank no. PQ373831) was successfully isolated from a diarrhoeal pig farm in Shanxi Province, China, being identified and kept at our laboratory. The LLC-PK1 cells were seeded overnight at 37 °C in an atmosphere with 5% CO_2_. Virus isolation and the pathogenicity and biological characteristics of the PDCoV strain were reported in our previous study [29]. The small-molecule drugs used in this study are as follows: chloroquine (CQ, HY-17589A, MCE), ferrostatin-1 (Fer-1, HY-100579, MCE), and liproxstatin-1 (Lip-1, HY-12726, MCE).

### TCID_50_ assay

Viral titers were determined by 50% tissue culture infectious dose (TCID_50_) assays in LLC-PK1 cells cultured in 96-well plates, according to the procedure described in our previous study [29]. Briefly, cells were washed and inoculated with tenfold serial dilutions of PDCoV in 100 μL of DMEM containing 10 μg/mL trypsin. After 2 h, the inoculum was removed, and the cells were washed before adding 200 μL of DMEM supplemented with 10% FBS and 1% penicillin–streptomycin. Cytopathic effect (CPE) was monitored daily for 3–5 days, and the TCID_50_ was calculated using the Reed–Muench method.

### Immunofluorescence assay

PDCoV-infected LLC-PK1 cells, along with uninfected LLC-PK1 cells, were fixed in 4% paraformaldehyde (Solarbio, Beijing) for 2 h. Subsequently, the cells were permeabilized with 0.1% Triton X-100 (Sigma-Aldrich) for 10 min and blocked with 10% skim milk for 1 h. Following this, the cells were incubated overnight with mouse antiserum specific to the PDCoV N protein, diluted at 1:500. The cells were incubated with secondary antibodies (Alexa Fluor™ Plus 594) for 1 h, followed by staining of the nuclei using 4′,6-diamidino-2-phenylindole (DAPI; Sigma-Aldrich, Merck, China). Then, the cells were examined using a confocal laser scanning microscope (Leica, Germany).

### Western blotting assay

Cells were lysed for 30 min on ice-cold lysis buffer (Solarbio, Beijing). Then, equal amounts of protein were separated on a 12% sodium dodecyl sulfate (SDS)-polyacrylamide gel electrophoresis (PAGE) gels and transferred to polyvinylidene fluoride (PVDF) membranes (Merck-Millipore, USA). After blocking with 10% nonfat milk, the membranes were incubated with primary antibodies, followed by horseradish peroxidase (HRP)-conjugated secondary antibodies (EASYBIO, Beijing). These primary antibodies included the PPRV N protein from our laboratory, anti-LC3 polyclonal antibody (Proteintech,14600-1-AP), anti-P62/SQSTM1 polyclonal antibody (Proteintech. 18420-1-AP), and anti-β-actin antibody (Proteintech, 20536-1-AP) from Proteintech Group, Inc. Protein bands were detected using Pierce enhanced chemiluminescence (ECL) western blotting substrate (Thermo Fisher Scientific), and the band density was quantified using ImageJ software (version 1.38).

### Transcriptome analysis

LLC-PK1 cells were infected with PDCoV at a multiplicity of infection (MOI) of 2. Meanwhile, mock-infected cells served as a control. At 1.5 and 18 h postinfection, PDCoV-infected LLC-PK1 cells were harvested using TRIzol reagent (Invitrogen, Shanghai, China) and were submitted to Beijing Novogene Co., Ltd for RNA extraction and sequencing. RNA integrity was assessed using the RNA Nano 6000 assay kit of the Bioanalyzer 2100 system (Agilent Technologies, CA, USA). mRNA was isolated from total RNA using poly(A) selection with magnetic beads and then chemically fragmented. The fragmented mRNA was used as a template to synthesize double-stranded cDNA. This involves first-strand synthesis with a random hexamer primer and reverse transcriptase, followed by second-strand synthesis. The cDNA fragments undergo end repair, A-tailing, and ligation of sequencing adapters to their ends. The library is size-selected using magnetic beads and then polymerase chain reaction (PCR)-amplified with primers that add unique index sequences for sample multiplexing. Raw data (raw reads) were screened and identified according to Q20, Q30, and GC content. The reference genome was built and paired-end clean reads were aligned to the reference genome using Hisat2 (version 2.0.5). The mapped reads of samples were assembled by StringTie (version 1.3.3b). Feature Counts (version 1.5.0-p3) was used to count the read numbers mapped to each gene and fragments per kilobase million (FPKM) was calculated on the basis of the length and reads count of the gene. *p*_adj_ represents the adjusted *p*-value. The statistical model calculated the probability for hypothesis testing (*p*-value), and genes with |log_2_(FoldChange)|> 0 and DESeq2 *p*-value ≤ 0.05 were considered DEGs.

### Proteomic analysis

LLC-PK1 cells were infected with PDCoV at an MOI of 2. Meanwhile, the mock-infected cells served as a control. At 18 h postinfection, PDCoV-infected LLC-PK1 cells were harvested using TRIzol reagent (Invitrogen, Shanghai, China) and submitted to Beijing Novogene Co., Ltd for protein extraction and sequencing. The sample was lysed using DB lysis buffer (8 M urea, 100 mM triethylammonium bicarbonate (TEAB), pH 8.5), followed by ultrasonication on ice for 5 min. After centrifugation for 15 min, the supernatant was collected. Dithiothreitol (DTT, 1 M) was added and incubated at 56 °C for 1 h to reduce disulfide bonds. Then, alkylation was performed by adding an adequate amount of iodoacetamide, followed by incubation in the dark at room temperature for 1 h and then on ice for 2 min. The experiment used Tandem Mass Tags (TMT) technology by Thermo Scientific (Waltham, MA, USA) for quantitative proteomics analysis. The ratio of the mean quantification values from all biological replicates for each protein in the comparison sample pair was used to determine the fold change (FC). The proteins whose quantitation significantly different between experimental and control groups (*p*-value < 0.05 and FC > 1.2 or FC < 0.83) were defined as DEPs.

### Enrichment analysis of DEGs and DEPs

Gene Ontology (GO) and Kyoto Encyclopedia of Genes and Genomes (KEGG) pathway enrichment analysis of DEGs was implemented by using the cluster Profiler R package [30]. For DEPs, GO, transcription factors (TF), and InterPro (IPR) functional analysis were conducted using the interproscan program against the nonredundant protein database, and the databases of Clusters of Orthologous Groups (COG) and KEGG were used to analyze the protein family and pathway. The cell mPLOC 2.0 website [31] was used to annotate the subcellular localization information. The genes were ranked by gene set enrichment analysis (GSEA) [32].

### Protein–protein interaction network analysis

The protein–protein interactions (PPI) were predicted using the STRING-db server [33]. The PPI of the transcriptomics were extracted with a score of >0.9, and a score of >0.4 for proteomics. The obtained network was visualized using Cytoscape software (Version v.9.1) with Molecular Complex Detection (MCODE) analysis with default parameters (Degree Cutoff = 2, Node Score Cutoff = 0.2, K-Core = 2, Max. Depth = 100) [34].

### Integrated analysis of transcriptomic and proteomic

The Spearman rank correlations (*r*) and their corresponding *p*-values were calculated using R statistical software. The analysis parameters were set to include the log_2_(FC) values of mRNA transcripts alongside their associated proteins. Comparative analysis of the transcriptome and proteome was conducted to identify commonly enriched GO terms, KEGG pathways, GSEA, and PPI.

### RNA interference

siRNAs targeting ATG5 (si-ATG5) (target site: GCUUCGAGAUGUGUGGUUUTT) were designed and synthesized by Tsingke, Inc. (Beijing, China). At 60% confluence, the siRNAs were transfected into the cells using the transfection reagent JetPRIME (Polyplus) according to the manufacturer’s protocol and then infected with PDCoV (MOI = 2) for 24 h. Then, cells were collected for Western blotting.

### Ferrous iron assays

The FerroOrange probe was used for fluorescence imaging of intracellular Fe^2+^. Cells were infected with PDCoV (MOI = 2) for 24 h. FerroOrange (1 µM) dispersed in serum-free medium was added to the cells, followed by incubation for 30 min at 37 °C. Cells were photographed under a fluorescent microscope (Leica, Germany). The fluorescence intensity was analyzed using ImageJ software (version 1.38).

### Quantification of lipid-ROS

For lipid- reactive oxygen species (ROS), cells were first infected with PDCoV (MOI = 2) for 24 h and were incubated with 10 µM of C11-4,4-difluoro-4-bora-3*a*,4*a*-diaza-*s*-indacene (BODIPY) for 30 min at 37 °C, 5% CO_2_ protecting from light. Excess C11-BODIPY was removed by washing the cells with phosphate-buffered saline (PBS). The cells were washed with PBS and were photographed under a fluorescent microscope (Leica, Germany). The fluorescence intensity was analyzed using ImageJ software.

### MDA assays

The lipid peroxidation levels were measured using a colorimetric reaction based on the reaction of malondialdehyde (MDA) and thiobarbituric acid (TBA) to produce a red product. Cells were infected with PDCoV (MOI = 2) for 24 h. Lipid peroxidation MDA assay kit (Beyotime, S0131S) instructions were followed exactly, and cell lysates from different treatments were mixed with MDA assay solution at 100 °C for 15 min, cooled to room temperature, and centrifuged at 1000 × *g* for 10 min. The supernatant was collected, and absorbance was measured at 532 nm using a microplate reader.

### Statistical analysis

Statistical analyses were performed using an independent-samples *t*-test or two-way analysis of variance (ANOVA) followed by Tukey’s multiple-comparisons test. All data are presented as mean ± standard deviation (SD) from at least three independent experiments, and statistical computations were carried out with GraphPad Prism software (version 8.0.2). A *p*-value < 0.05 was considered statistically significant.

## Results

### Transcriptomic analysis of PDCoV-infected LLC-PK1 cells

**Figure 1:**
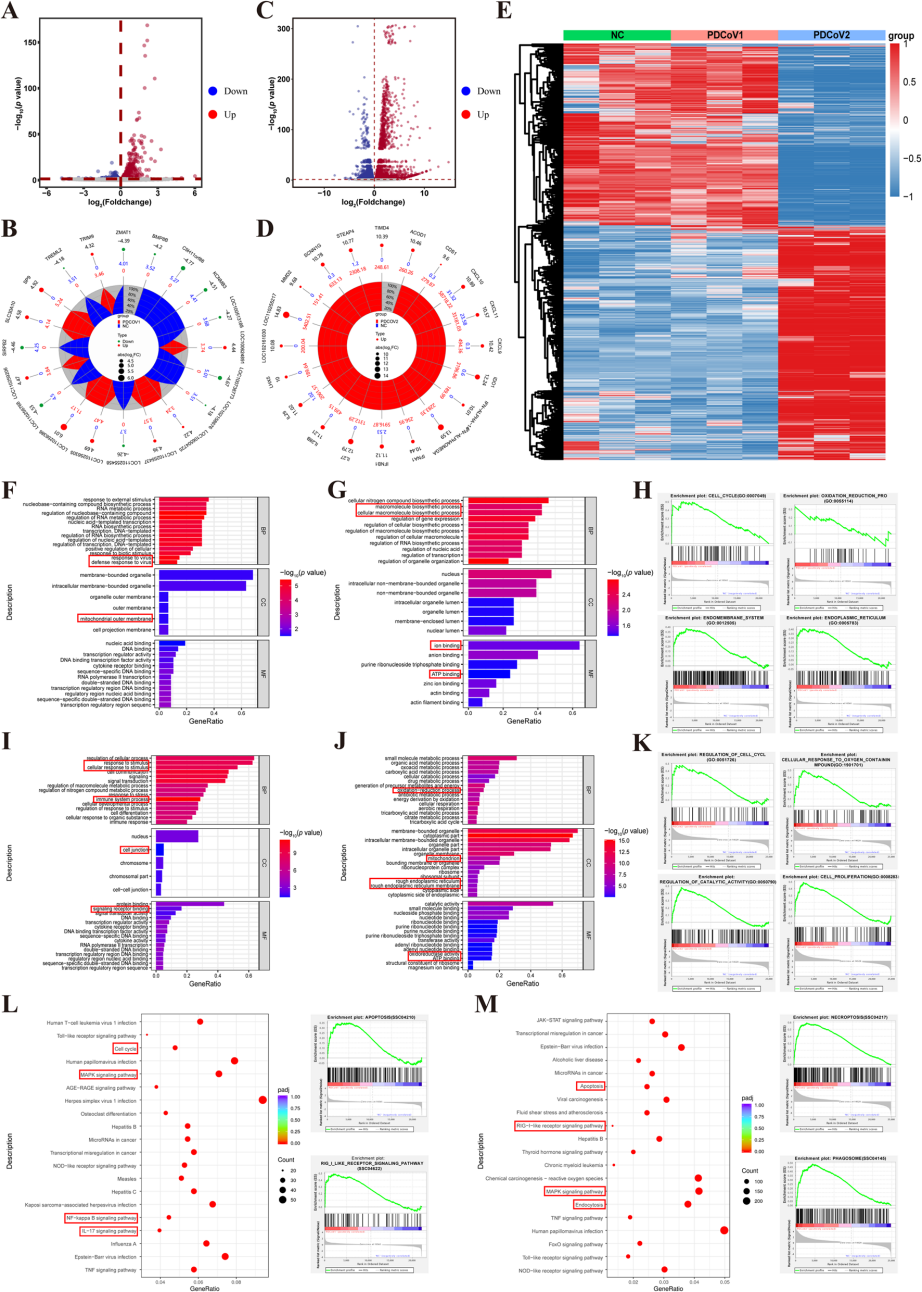
**Transcriptome sequencing analysis of the DEGs in PDCoV-infected LLC-PK1 cells**. **A**, **C** Volcano diagram of DEGs at 1.5 h post-infection and 18 h post-infection. Significantly upregulated genes are shown as *red dots*. Significantly downregulated genes are shown as *blue dots*. Nonsignificant DEGs are shown in *gray*. **B**, **D** Radar map of the top 20 significant DEGs with |log_2_(FC)| in PDCoV1 and PDCoV2 groups, respectively. *Circles* from outside: (1) gene name, (2) log_2_(FC), (3) the expression abundance in NC group, (4) the expression abundance in PDCoV group. Up- and downregulated genes are shown in *red* and *green*. A value of 0 represents an extremely small quantity, essentially zero. **E** Heatmap showing the expression levels of the differentially expressed genes. Columns represent individual samples, and rows indicate genes with significant expression differences between the different groups. Expression levels in the heatmaps are coded from *blue* (low) to *red* (high). NC group (*green*) is PDCoV-uninfected LLC-PK1 cells. PDCoV1 group (*pink*) is PDCoV-infected LLC-PK1 cells at 1.5 h post-infection. PDCoV2 group (*blue*) is PDCoV-infected LLC-PK1 cells at 18 h post-infection. **F**–**K** GO annotations of DEGs. Top 15 enriched GO terms for upregulated (**F**) and downregulated DEGs (**G**) for the PDCoV1 group. Top 15 enriched GO terms for upregulated (**I**) and downregulated DEGs (**J**) for the PDCoV2 group. **H**, **K** GSEA of enriched GO in PDCoV1 and PDCoV2 group. **L**, **M** The top 20 pathways identified by KEGG enrichment analysis of the DEGs and two screened pathways of GSEA in PDCoV1 and PDCoV2 groups, respectively. The color of *p*_adj_ (*p* value after correction for multiple hypothesis tests) is coded from *red* (low) to *blue* (high). Count is the number of DEGs annotated to the KEGG pathway.

Following PDCoV infection, the levels of viral infectivity were evaluated in LLC-PK1. In the PDCoV-uninfected (NC group) and PDCoV-infected LLC-PK1 cells (PDCoV group) at 1.5 h post-infection, no cytopathic effect (CPE) was observed (Additional file 1A). Obvious CPE, characterized by rounded, clustered cells with increased refraction, became evident at 18 h post-infection (Additional file 1A). The immunofluorescence assay (IFA) results were consistent with those of the CPE (Additional file 1B). Furthermore, the results of the transcriptomic quality indicated that clean reads were high quality and suitable for further analysis (Additional file 2). The expression values of all genes demonstrated that the samples were repeatable in NC and PDCoV groups (Additional file 3 A). A total of 1448 DEGs were identified in the PDCoV group at 1.5 h post-infection, comprising 829 upregulated genes and 619 downregulated genes (Figure 1A). Among the top 20 of all DEGs, *SP9* and *TRIM9* were significantly upregulated, whereas *ZMAT1* was significantly downregulated that may involve in the recognition, binding, and stability regulation of RNA (Figure 1B). Meanwhile, *RSAD2*, *DUSP6*, *OAS2*, *JUNB*, and *EGR1* were located in the top 10 DEGs that were significantly upregulated and may be involved in innate antiviral defense, cell cycle, and apoptosis (Additional file 4). Among the top 10 of downregulated DEGs, *PDK4*, *RNPC3*, *ARRDC3*, and *SKIDA* were identified, possibly associated with metabolism, cell motility, and signal transduction (Additional file 4). At 18 h post-infection, a total of 11,753 DEGs were identified, including 6615 upregulated and 5138 downregulated genes (Figure 1C). In the top 20 of all DEGs, DEGs related to the innate immunity, inflammatory responses were significantly upregulated, such as *IL27*, *IL29*, *IFNB1*, and *CXCL10* (Figure 1D). In the top 10 of the downregulated DEGs, *ANPEP*, *LRP2*, and *GPX1* were identified, which may be involved in viral infection, cell cycle, and ferroptosis signaling pathways (Additional file 5). Additionally, the heatmap data illustrated the difference in genes expression at 1.5 and 18 h post-infection (Figure 1E). These results suggest that PDCoV infection may induce innate antiviral defense and alter the cell cycle of LLC-PK1 cells.

### Enrichment analysis of DEGs in PDCoV-infected LLC-PK1 cells

To obtain further insights into the functions of DEGs, GO and KEGG analysis were conducted. On GO analysis, at 1.5 h post-infection, the upregulated DEGs were predominantly enriched in defense response to virus, mitochondrial outer membrane, and the downregulated DEGs were enriched in biosynthetic processes, ion binding, and adenosine triphosphate (ATP) binding (Figures 1F, G). GO analysis of all DEGs were significantly enriched in regulation of RNA metabolic process, nucleus, and outer membrane (Additional file 3B). The results of GSEA revealed that upregulated DEGs were enriched in cell cycle, oxidation reduction process, endomembrane system, and endoplasmic reticulum (Figure 1H). At 18 h post-infection, the upregulated DEGs were primarily involved in response to stimulus, immune system process, cell junction, as well as signaling receptor binding (Figure 1I). The downregulated DEGs were mainly enriched in oxidation–reduction process, mitochondrion, rough endoplasmic reticulum, and rough endoplasmic reticulum membrane (Figure 1J). Moreover, GO analysis of all DEGs were significantly enriched in cellular response to stress and intracellular membrane-bounded organelle (Additional file 3 C). The results of GSEA suggested that upregulated DEGs were significantly enriched in regulation of cell cycle, cellular response to oxygen containing compound, regulation of catalytic activity, and cell proliferation in the PDCoV group (Figure 1K).

On KEGG and GSEA analysis of all DEGs, the results revealed that cell cycle, MAPK, nuclear factor (NF)-kappa B, interleukin (IL)-17 pathways, apoptosis, and RIG-I-like receptor signaling pathway were enriched at 1.5 h post-infection (Figure 1L). We found that upregulated DEGs were enriched in MAPK, necroptosis, NF-kappa B, and IL-17 pathways, and downregulated DEGs were enriched in the cell cycle (Additional files 3D, E). At 18 h post-infection, all DEGs were mainly enriched in apoptosis, RIG-I-like receptor, MAPK, endocytosis, necroptosis, and phagosome (Figure 1M). Meanwhile, significantly upregulated DEGs were primarily involved in Janus kinase (JAK)/signal transducer and activator of transcription (STAT), RIG-I-like receptor, MAPK pathways, whereas downregulated DEGs were enriched in the cell cycle and fatty acid metabolism (Additional files 3 F, G). Notably, the autophagy pathway was ranked within the top 50 of KEGG pathways (Additional file 6).

### Integrated analysis of transcriptomic data at 1.5 and 18 h post-PDCoV infection in LLC-PK1 cells

**Figure 2:**
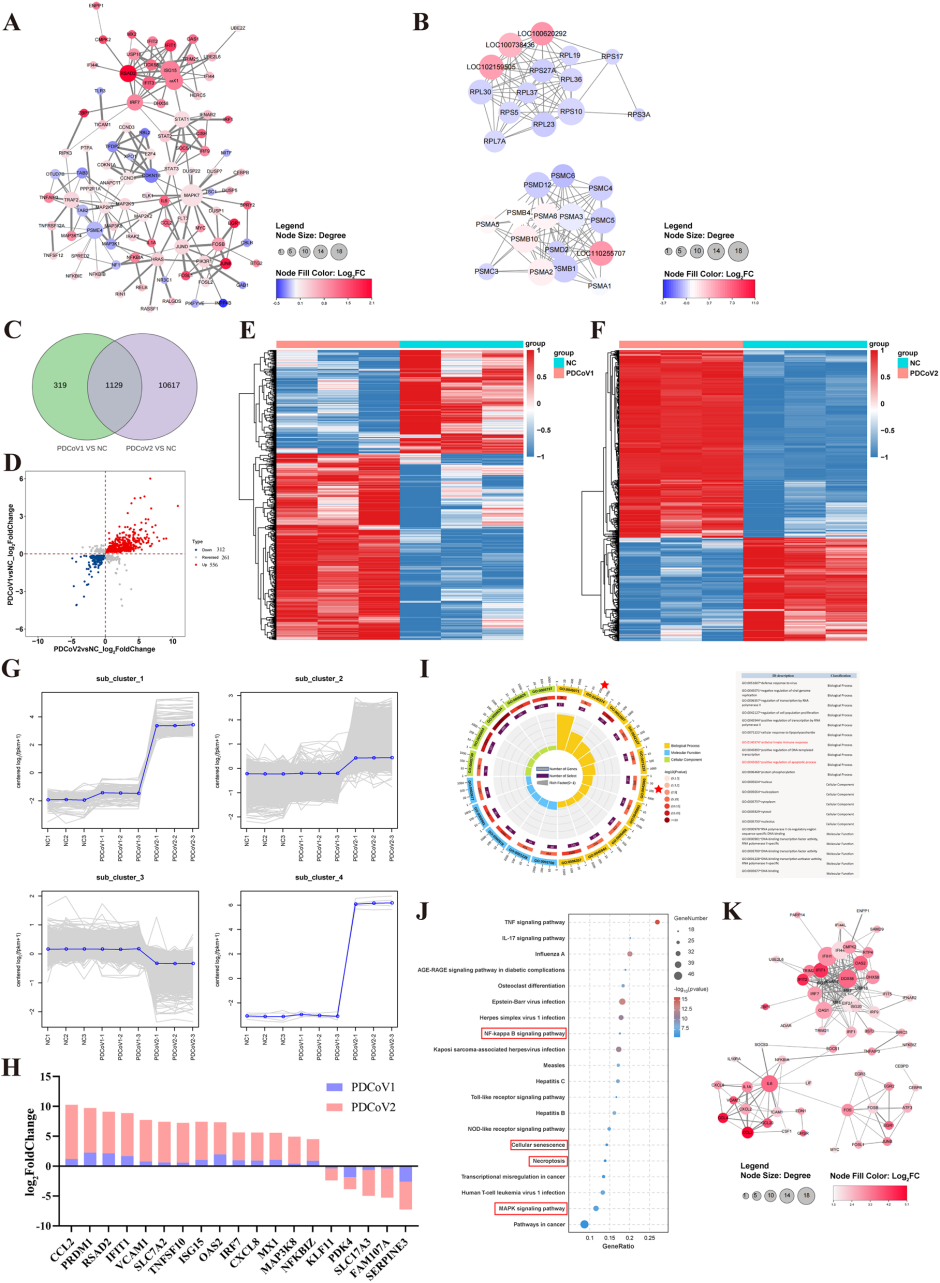
**Interaction analysis of DEGs in PDCoV-infected LLC-PK1 cells in PDCoV1 and PDCoV2 groups**. **A**, **B** PPI networks of the significantly upregulated (*red*) and downregulated (*blue*) DEGs at 1.5 h post-infection and 18 h post-infection, respectively. The size of the circles and the depth of the colors indicate the numbers of interacted proteins and the fold change. **C** Venn diagram illustrating the numbers of DEGs in the PDCoV1 group (*green*) and PDCoV2 group (*purple*). **D** Four-quadrant diagrams of the common DEGs expressed in two PDCoV groups. The expression levels of DEGs exhibited as consistent are shown as *red* (upregulated) and *blue* (downregulated). The expression levels of DEGs exhibited as converse are shown in *gray*. **E**, **F** Heatmap of the common DEGs in PDCoV1 and PDCoV2 group. **G** Four unique clusters of DEGs expression trends from 1.5 to 18 h post-infection. The horizontal axis represents individual samples, and the vertical axis represents gene expression. **H** Histogram of the DEGs. **I** Circle maps of the common DEGs that were classified by the GO database. *Circles* from outside: (1) scale, (2) GO categories and different color-marked biological process (*yellow*), cellular component (*green*), and molecular function (*blue*), (3) bar graph of the total DEGs numbers, where the gradient represents the *p*-value of the enriched term, (4) bar graph of the significantly upregulated DEGs (*purple*), (5) the enrichment factor for each GO term (*left*) and their GO function (*right*). **J** The top 20 pathways identified by KEGG enrichment analysis of the common DEGs. The color of the −log_10_ (*p* value) is coded from *blue* (low) to *red* (high). Gene Number is the number of DEGs annotated to the KEGG pathway. **K** PPI networks of the significantly upregulated (*red*) DEGs expressed in both PDCoV groups.

To examine the interactions among the DEGs at 1.5 or 18 h post-infection, we constructed PPI networks based on the high-degree proteins (Figures 2A, B). At 1.5 h post-infection, upregulated RADS2, IFIT1, and MAPK7 along with the downregulated CDKN1B and PSME4 were central in the network, being involved in innate antiviral defense and cell cycle regulation (Figure 2A). At 18 h post-infection, for all DEGs, we identified that significantly upregulated proteasome subunit (PMS)A6, PMSB10, and PMSA2, and downregulated ribosomal protein small subunit (RPS) and ribosomal Protein large subunit (RPL) family proteins were central in the network, being associated with immune responses, cell proliferation, and stress responses of host cells (Figure 2B). Moreover, we found that significantly upregulated ISG15, IFIT1, and heat shock protein 90kDa beta member 1 (HSP90B1) were prominent interaction partners, which represent key cellular defense and surveillance systems (responding to interferon signaling, recognizing foreign nucleic acids, and maintaining endoplasmic reticulum homeostasis) at 18 h post-infection (Additional file 3H). Meanwhile, significantly downregulated integrin subunit alpha 1 (ITGA1) and Sonic Hedgehog (SHH) emerged as significant interaction partners, which were related to cell–extracellular matrix adhesion and proliferation at 18 h post-infection (Additional file 3I).

To further investigate the genetic correlations at different time points, a total of 1129 common DEGs were assessed (Figures 2C, D). The heatmap data illustrated the differential expression of these genes at 1.5 and 18 h post-infection, respectively (Figures 2E, F). On the basis of the expression levels of all genes, these genes were categorized into four subclusters (Figure 2G). In subclusters 1, 2, and 4, genes were downregulated in NC and PDCoV groups at 1.5 h post-infection, while they exhibited varying degrees of upregulation at 18 h post-infection, such as *CCL2*, *IFIT2*, *IL29*, *IL7R*, *CXCL10*, and *IFNB1* (Figure 2G and Additional file 7). In subcluster 3, genes were upregulated in NC group and PDCoV groups at 1.5 h post-infection, but they were downregulated at 18 h post-infection, such as *ANPEP* and *FAM107A* (Figure 2G and Additional file 7). On the basis of gene expression of PDCoV2 group (log_2_FoldChange)/PDCoV1 group (log_2_FoldChange) being more than 1.0, we screened *TNFSF10*, *ISG15*, *IFIT1*, *OAS2*, *SLC7A2*, *IRF7*, *MX1*, and *FAM107A*, which relate to host cell immunity, antiviral responses, and cell growth (Figure 2H). Furthermore, GO analysis indicated that common DEGs were primarily involved in antiviral innate immune responses and positive regulation of apoptotic process (Figure 2I). KEGG analysis showed that the common DEGs were enriched in NF-kappa B, cell senescence, necroptosis, and MAPK signaling pathways (Figure 2J). The results of PPI network showed that IFIT1, DEAD-box helicase 58 (DDX58), OAS2, IL6, C–C motif chemokine ligand 4 (CCL4), and early growth response 1 (EGR1) were more common interaction partners (Figure 2K). Therefore, these data suggest that the inflammation, antiviral innate immunity responses, autophagy, and cell cycle of LLC-PK1 cells were activated upon PDCoV infection.

### Proteomic analysis of PDCoV-infected LLC-PK1 cells at 18 h post-infection

**Figure 3:**
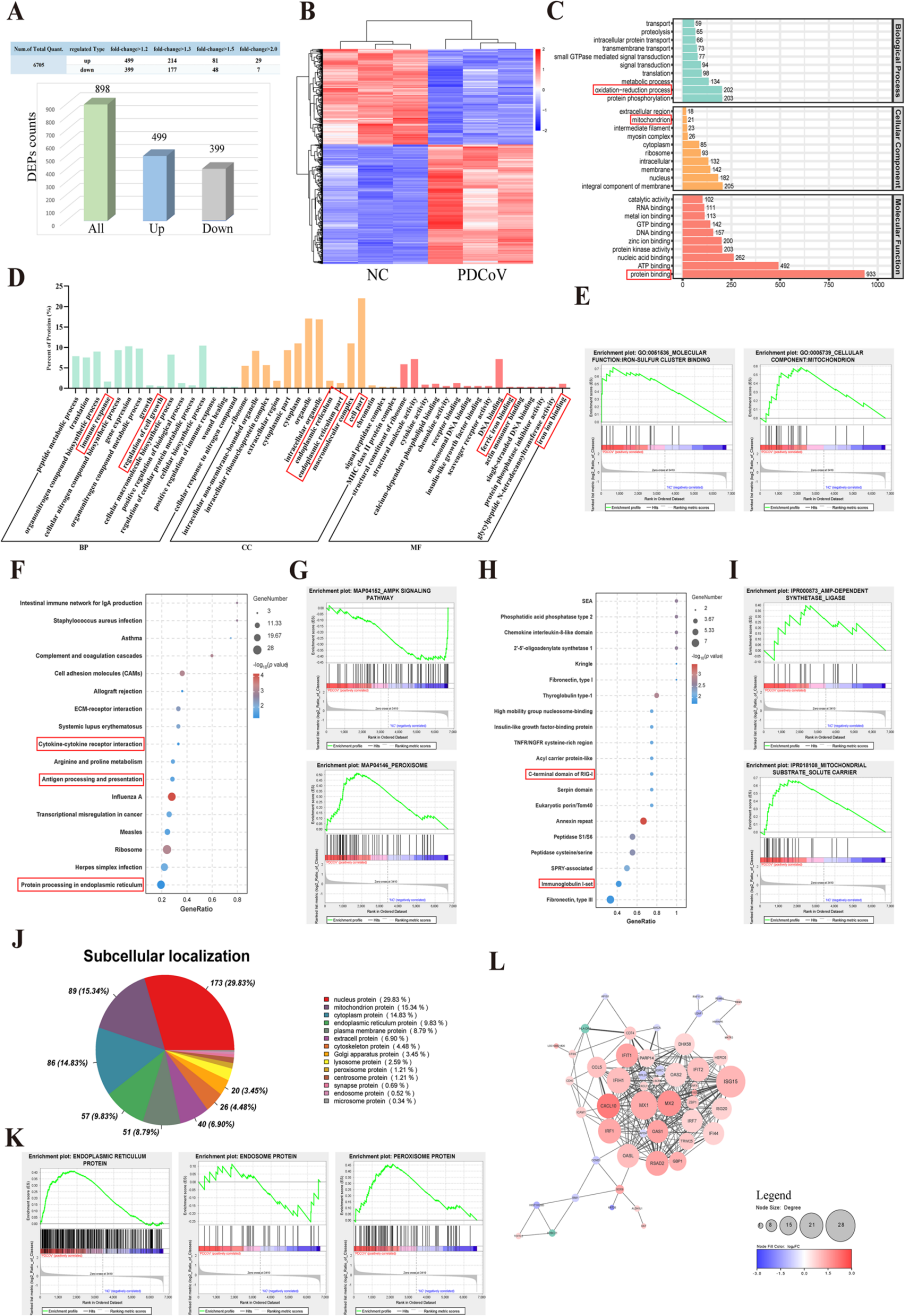
**Proteome analysis of the DEPs in PDCoV-infected LLC-PK1 cells at 18 h post-infection**. **A** DEPs determined by* p*-value < 0.05 and different FC and the histogram of the significant DEPs with the numbers. Up- and downregulated genes are shown in *blue* and *gray*. All the significantly DEPs are shown in *green*. **B** Heatmap of DEPs. Expression levels in the heatmaps are color coded from *blue* (low) to *red* (high). **C** GO annotations of all identified proteins. **D**, **E** Top 16 of GO analysis and GSEA of enriched GO for DEPs. **F** KEGG enrichment analysis of DEPs. The color of −log_10_ (*p* value) is coded from *blue* (low) to *red* (high). Gene Number is the number of DEPs annotated to the KEGG pathway. **G** GSEA of enriched KEGG. **H** The top 20 of IPR enrichment analysis. The color of −log_10_(*p* value) is coded from *blue* (low) to *red* (high). Gene Number is the number of DEPs annotated to IPR. **I** GSEA of enriched IPR. **J** Subcellular localization composition of DEPs. Different color represents different subcellular localization. **K** GSEA of the subcellular localization. **L** PPI networks of the significantly upregulated (*red*) and downregulated (*blue*) DEPs. The size of the *circles* indicated the numbers of interacting proteins.

To assess the quality of the identified proteins, we evaluated several parameters, including principal components analysis, the coefficient of variation, peptide lengths, near-zero distribution of mass error, unique peptides, coverage, and molecular weight (Additional file 8A–G). These results offer a comprehensive overview of the proteins present in PDCoV-infected LLC-PK1 cells and indirectly indicate the high quality of our results, which are suitable for further analysis. As for DEPs, 499 upregulated and 399 downregulated DEPs were identified (Figure 3A). Hierarchical clustering was conducted to illustrate the expression patterns of the differential proteome (Figure 3B). In the top 20 of all DEPs, IFIT2, OAS1, RSAD2, CXCL10, and IRF1, were significantly upregulated being associated with viral invasion resistance, immune response, and inflammatory response (Additional file 9). Meanwhile, keratin 20 (KRT20) was significantly downregulated, being related to maintaining the structure and mechanical stability of epithelial cells (Additional file 9).

To obtain further insights into the functions of all identified proteins, GO, eukaryotic orthologous groups (KOG), TF, KEGG, and IPR domains were annotated. GO analysis indicated that all identified proteins were primarily associated with protein oxidation–reduction, mitochondrion, and protein binding (Figure 3C). The KOG classification revealed that proteins were related to cell cycle and signal transduction mechanisms (Additional file 8H). The TF analysis indicated a predominance of zf-C2H2, ZBTB, HMG, MYB, and RHD annotations, which are primarily linked to transcriptional regulation, inflammation, and immunity (Additional file 8I). KEGG analysis suggested that cell growth and organismal systems were predominantly enriched (Additional file 8 J). Additionally, IPR of the identified proteins was mainly enriched in protein kinase domains and RNA recognition motif domains (Additional file 8K). For all DEPs, GO analysis revealed that immune response, regulation of cell growth, endoplasmic reticulum, cell part, ferric iron binding, and iron ion binding were mainly enriched (Figure 3D). Among these findings, the upregulated DEPs were primarily involved in immune response, defense response, lipid binding, receptor binding, and iron-sulfur cluster binding (Additional file 10 A). The downregulated DEPs were enriched in regulation of cell growth and protein binding (Additional file 10B). GSEA results revealed that iron-sulfur cluster binding and mitochondrion were significantly enriched in the PDCoV group at 18 h post-infection (Figure 3E). KEGG analysis of all DEPs indicated that antigen processing and presentation, cytokine–cytokine receptor interaction, and protein processing in endoplasmic reticulum were mainly enriched (Figure 3F). Among them, the upregulated DEPs were enriched in antigen processing and presentation, RIG-I-like receptor signaling pathway, and necroptosis, whereas the downregulated DEPs were enriched in the AMPK and PI3K–Akt signaling pathways (Additional file 10C, D). GSEA results revealed that AMPK signaling pathways and peroxisome were enriched (Figure 3G). Furthermore, for DEPs, the top 20 of the IPR annotation were mainly enriched in C-terminal domain of RIG-1 and immunoglobulin 1 set (Figure 3H). GSEA of IPR indicated that adenosine monophosphate (AMP)-dependent synthetase and mitochondrial substrate solute carrier were enriched (Figure 3I). The results of subcellular localization indicated that DEPs were distributed in nucleus (29.83%), mitochondrion (15.34%), and cytoplasm (14.83%) (Figure 3J). GSEA of subcellular localization indicated that DEPs mainly involved in endoplasmic reticulum protein, endosome protein, and peroxisome protein (Figure 3K). PPI networks were constructed to investigate the relationships of DEPs, which indicated that CXCL10, MX2, ISG15, RSAD2, OASL, IRF1, and IFIT1 were significant interactions (Figure 3L). Our results indicate PDCoV-infected LLC-PK1 cell changes in innate immunity, inflammatory, antiviral defense, autophagy, and ferroptosis.

### Integrated analysis of DEGs and DEPs in PDCoV-infected LLC-PK1 cells at 18 h post-infection

**Figure 4:**
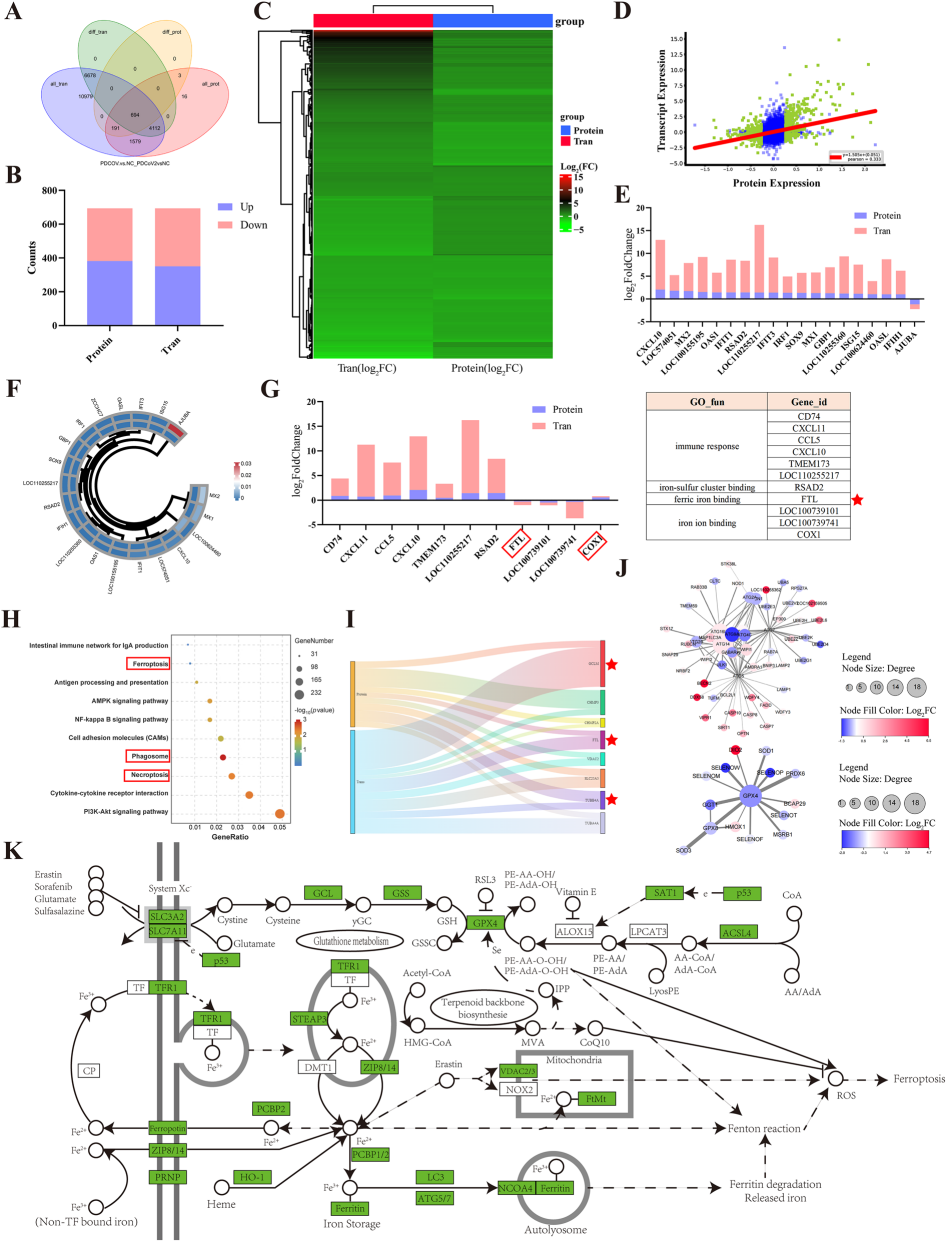
**Integrative transcriptomics and proteomics analysis of PDCoV-infected LLC-PK1 cells at 18 h post-infection**. **A** Venn diagrams illustrating the numbers of all the identified genes (*purple*), differentially expressed genes (*green*), all proteins (*pink*), and differentially expressed proteins (*yellow*). **B** Histogram of the common genes and proteins. The *x*-axis represents the proteomics (Protein) and transcriptomics (Tran), and the *y*-axis shows the numbers of identified DEGs and DEPs. The upregulated genes are shown in *purple*, and the downregulated genes are shown in *pink*. **C** The clustering analysis of the identified 694 genes/proteins at the mRNA and protein levels. Expression levels of mRNA and protein levels are coded from *green* (low) to *red* (high). **D** Correlation analysis of transcriptome and proteome expression levels. The *green dots* represent proteins with significantly different expression, and the *blue* represents proteins with no significantly different expression. **E** Histogram of the DEGs/DEPs based on log_2_ (FC) in transcriptomics group/log_2_ (FC) in proteomics group > 1. **F** Heatmap of *p* value of the DEGs/DEPs based on log_2_ (FC) in transcriptomics group/log_2_ (FC) in proteomics group > 1. *p* value is coded from *blue* (low) to *red* (high). **G** GO analysis related to immune response, iron-sulfur, and ferric iron binding. **H** Top 10 of KEGG analysis of the DEGs/DEPs. The color of −log_10_ (*p* value) is coded from *blue* (low) to *red* (high). **I** Distribution map of DEGs/DEPs associated to ferroptosis, necroptosis, and phagosome at the mRNA and protein levels. **J** PPI networks of DEGs/DEPs associated to autophagy and ferroptosis. The significantly upregulated and downregulated DEGs/DEPs are shown in *red* and *blue*. The size of the circles and the depth of the colors indicate the numbers of interacted proteins and the fold change. **K** Mapping of DEGs and DEPs to KEGG pathways (ferroptosis). The *squares* represent genes, and *green* indicates regulated gene/protein at the mRNA and protein levels.

To determine the complementarity and integration of mRNAs and proteins, we analyzed the integration analysis of the DEGs and DEPs. A total of 11,484 DEGs and 888 DEPs were identified at 18 h post-infection, including 694 common genes/proteins at 18 h post-infection (Figure 4A). In common genes/proteins, 351 DEGs and 382 DEPs were upregulated, while 343 DEGs and 312 DEPs were downregulated (Figure 4B). These results are represented as heatmaps (Figure 4C). Furthermore, we analyzed the distribution of the ratios of mRNA to protein, showing green dots for common genes/proteins (Figure 4D). On the basis of log_2_ (FoldChange) > 1 and *p* value <0.05 of the common genes or proteins in proteome and transcriptome, we found that CXCL10, MX2, IFIT3, GBP1, and OASL were upregulated and AJUBA was downregulated, being involved in immune responses, antiviral defense, and cell regulation (Figures 4E, F). Meanwhile, in the genes/proteins with Tran-log_2_(FoldChange)/Protein-log_2_(FoldChange) > 1, CD47, CXCL11, CXCL10, TMEM173, RSAD2, FTL, and COX1 were screened, being related to inflammatory response, immune response, ferroptosis, and mitophagy (Figure 4G). GO analysis of these screened genes/proteins indicated that the immune response, iron-sulfur cluster binding, ferric iron binding, and iron ion binding were enriched (Figure 4G). KEGG analysis indicated that screened DEPs or DEGs were enriched in ferroptosis, AMPK, necroptosis, and phagosome during PDCoV infection (Figure 4H). Moreover, the DEGs or DEPs related to ferroptosis, necroptosis, and phagosome are listed in Table 1 and Figure 4I. PPI networks were constructed and suggested that the relationships of the significantly upregulated and downregulated DEPs associated with autophagy and ferroptosis, respectively (Figure 4J). The screened DEPs or DEGs related to the ferroptosis pathway were labeled by integration analysis of transcriptome and proteome in our study (Figure 4K).

**Table 1:** **DEGs and DEPs related to ferroptosis, necroptosis, and phagosome in integrated analysis of transcriptome and proteome**

KEGG	Gene ID	Tran ID	Prot ID	Protein (log_2_FC)	Protein (*p* value)	Tran (log_2_FC)	Tran (*p* value)
Ferroptosis (map04216)	GCLM	100153977	XP_001926413.2	−0.35855	0.003239342	−2.1151	3.37 × 10^−95^
Necroptosis (map04217)	CHMP3	100522249	XP_003354782.3	−1.00605	8.44082 × 10^−5^	−0.31743	0.000488
	CHMP2A	100622139	XP_020953074.1	0.297458	0.001595081	−0.18854	0.033484
	FTL	397035	NP_001231060.1	−0.27625	0.002746787	−0.70861	2.29 × 10^−42^
	VDAC2	397659	NP_999534.1	0.292355	0.008042266	−0.40973	1.77 × 10^−11^
	SLC25A5	100158185	XP_001927475.1	0.382885	0.021483783	0.616703	7.50 × 10^−30^
Phagosome (map04145)	TUBB4A	100736584	XP_003480860.1	−0.60035	0.028755658	−0.37092	0.000177
	TUBA4A	100151951	XP_001928370.3	−0.30382	0.032751973	−0.81623	3.04 × 10^−29^

### PDCoV infection induces autophagy and ferroptosis in LLC-PK1 cells

**Figure 5:**
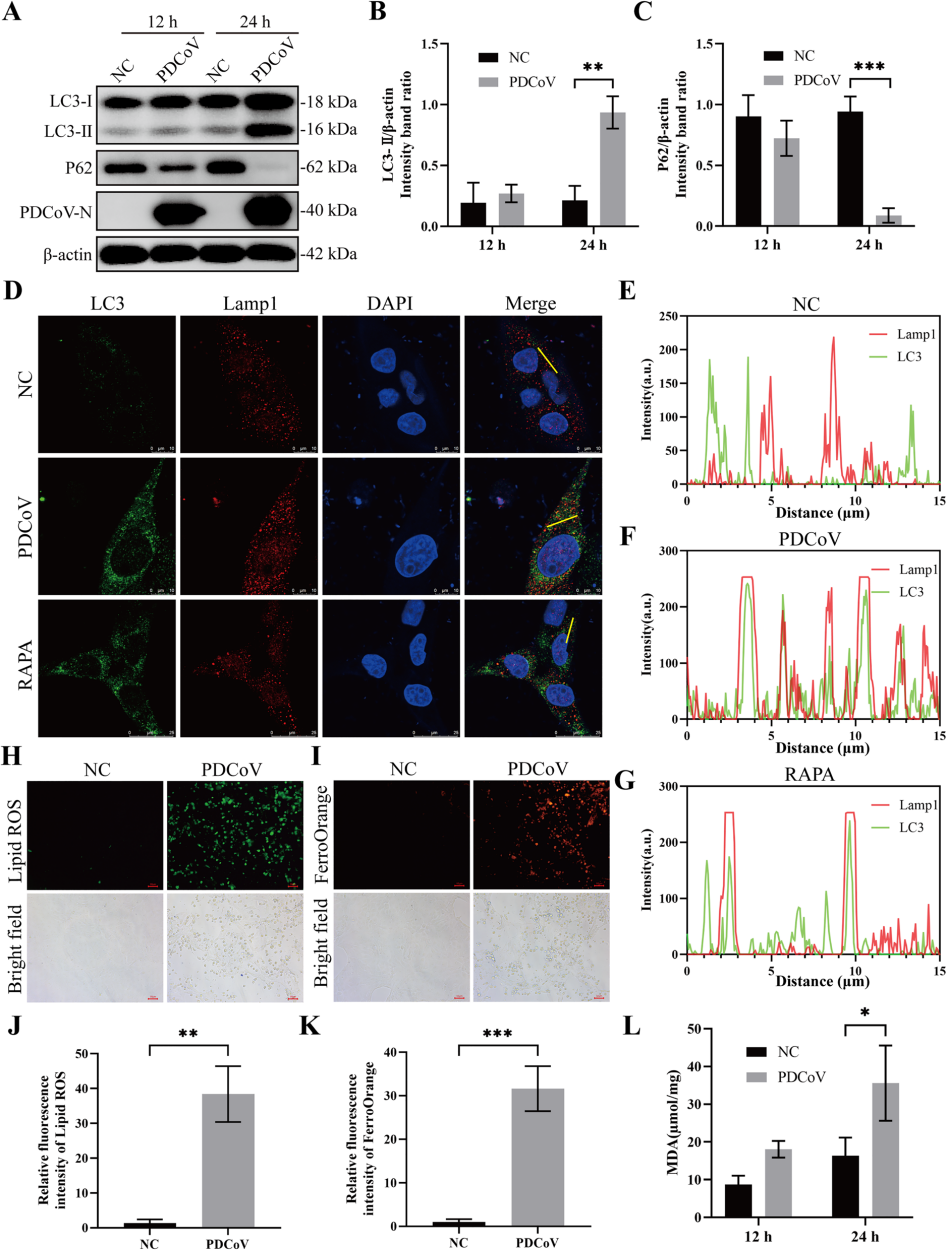
**PDCoV infection induces autophagy and ferroptosis in LLC-PK1 cells**. **A** LLC-PK1 cells were mock infected or infected with PDCoV (MOI = 2) at 12 and 24 h, then cell lysates were harvested and examined by Western blotting. The relative protein levels of LC3-II and P62 in **A** are shown in **B** and **C**. **D**–**G** Laser confocal microscopy analyses of the co-localization of LC3B and LAMP1 in LLC-PK1 cells. **D** LLC-PK1 cells were mock infected or infected with PDCoV (MOI = 2) for 24 h, and then the cells were fixed and processed for indirect immunofluorescence using antibodies against LC3B and LAMP1 proteins. Scale bars, 10 and 25 μm. Co-localization between LC3B and LAMP1 in panel **D** was analyzed by fluorescence intensity line measurement (*yellow line*) and is shown in panels **E**, **F**, and **G**. **H**–**L** Determination of the level of ferroptosis following PDCoV infection in LLC-PK1 cells. LLC-PK1 cells were infected as described above, then the cells were collected and subjected to determine the contents of lipid-ROS (**H**, **J**), ferrous ion (**I**, **K**), and MDA (**L**) in cells. Data are representative of three independent experiments and presented as mean ± standard deviation (SD). ***p < 0.001; **p < 0.01; *p < 0.05.

To further substantiate the above findings, we assessed the levels of autophagy and ferroptosis in PDCoV-infected LLC-PK1 cells. As a result, the levels of LC3-II and P62, two critical markers of autophagy, were significantly altered in LLC-PK1 cells following PDCoV infection, with an increase in LC3-II expression and a decrease in P62 expression in a time-dependent manner (Figures 5A–C). Furthermore, the distribution of LC3B exhibited typical punctate aggregation and demonstrated significant co-localization with lysosomal-associated membrane protein 1 (LAMP1), a lysosomal marker, in PDCoV-infected LLC-PK1 cells (Figures 5D–G), indicating the formation of autophagosomes and the induction of autophagic flux during PDCoV infection. Additionally, we evaluated the level of intracellular ferroptosis following PDCoV infection. The results revealed a significant elevation in the contents of lipid-ROS, a marker of ferroptosis, in LLC-PK1 cells infected with PDCoV (Figures 5H, J). Similarly, intracellular concentrations of ferrous iron (Fe^2+^) (Figures 5I, K) and malondialdehyde (MDA) (Figure 5L) were markedly increased, confirming that PDCoV infection induces ferroptosis in LLC-PK1 cells. These findings suggest that autophagy and ferroptosis are activated during PDCoV infection in LLC-PK1 cells.

### Inhibition of autophagy suppresses PDCoV-induced ferroptosis in LLC-PK1 cells

**Figure 6:**
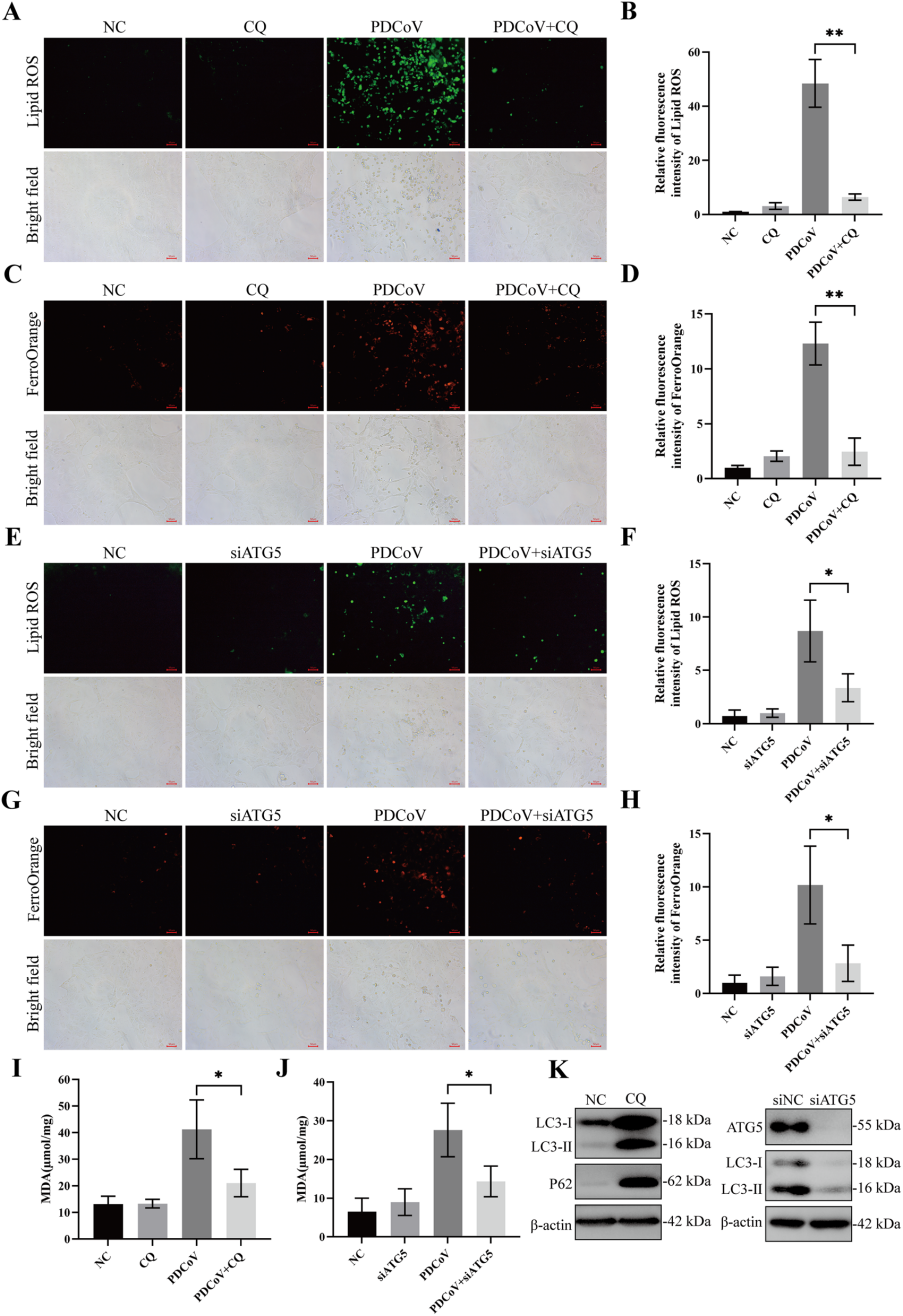
**Inhibition of autophagy suppresses PDCoV-induced ferroptosis in LLC-PK1 cells**. **A**–**D** LLC-PK1 cells were pretreated with CQ (50 µM) for 2 h followed by mock infection or PDCoV infection (MOI = 2). After adsorption for 2 h, the cells were further cultured in the presence or absence of CQ. Twenty-four hours after PDCoV infection, the cells were evaluated to determine the contents of lipid-ROS (**A**) and ferrous iron (**C**). The relative fluorescence levels of lipid-ROS and ferrous iron are shown in panels **B** and **D**. **E**–**H** LLC-PK1 cells were transfected with nonspecific control siRNA (siNC) or ATG5 siRNA (siATG5) for 24 h and then infected with PDCoV (MOI = 2) for 24 h. After infection, the cells were subjected to determine the contents of lipid-ROS (**E**) and ferrous iron (**G**). The relative fluorescence levels of lipid-ROS and ferrous iron are shown in panels **F** and **H**. **I** LLC-PK1 cells were pretreated and infected as described in panel **A**, then the cells were collected and subjected to determine the contents of MDA. **J** LLC-PK1 cells were pretreated and infected as described in **E**, then the cells were collected and subjected to determine the contents of MDA. **K** The inhibitory effects of CQ and siATG5 on autophagy were assessed using western blotting. Data are representative of three independent experiments and presented as mean ± standard deviation (SD). ***p < 0.001; **p < 0.01; *p < 0.05.

Given that PDCoV infection induces autophagy and ferroptosis in LLC-PK1 cells, we further explored the intrinsic relationship between these two processes. Therefore, we investigated the effect of autophagy inhibition on ferroptosis induced by PDCoV infection using CQ, an autophagy inhibitor. Our findings revealed that CQ treatment significantly reduced the upregulation of intracellular lipid-ROS (Figures 6A, B) and Fe^2+^ (Figures 6C, D) caused by PDCoV infection compared with controls, and it also alleviated the substantial increase in MDA content induced by PDCoV infection (Figure 6I). This preliminary evidence suggests that inhibition of autophagy suppresses PDCoV-induced ferroptosis. To strengthen these results, we employed siRNA targeting ATG5, a crucial protein for the initiation of autophagy, to inhibit autophagy followed by ferroptosis detection. Consistently, knockdown of ATG5 by RNA interference (RNAi) markedly decreased the intracellular levels of lipid-ROS (Figures 6E, F), Fe^2+^ (Figures 6G, H), and MDA (Figure 6J) induced by PDCoV infection in LLC-PK1 cells. Additionally, the inhibitory effects of CQ treatment and ATG5 knockdown on autophagy were confirmed by western blot analysis (Figure 6K). These results indicate that the induction of ferroptosis upon PDCoV infection is dependent on autophagy activation.

### Autophagy and ferroptosis are required for PDCoV replication in LLC-PK1 cells

**Figure 7:**
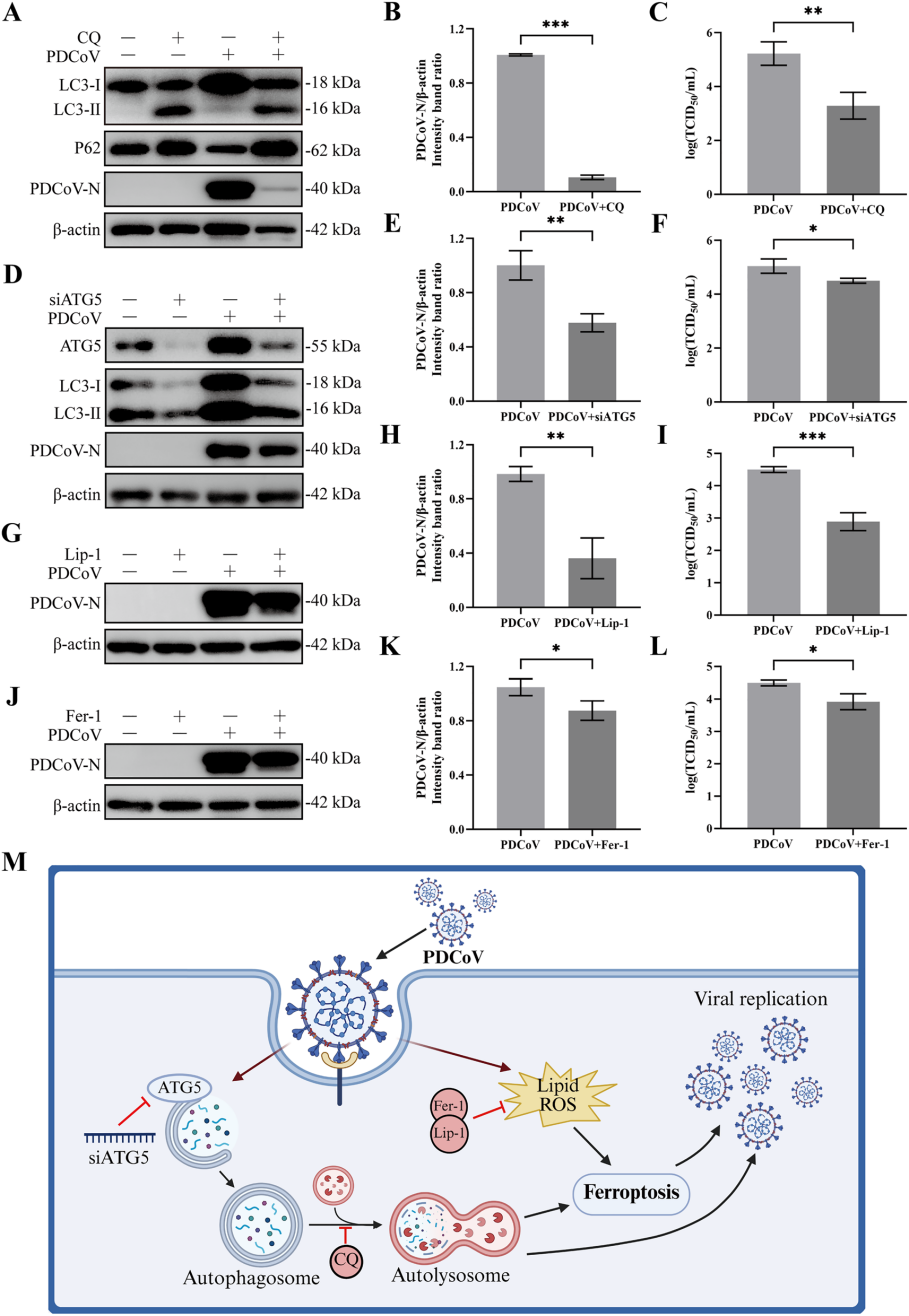
**Autophagy and ferroptosis are required for PDCoV replication in LLC-PK1 cells**. **A**–**F** Autophagy inhibition reduces PDCoV proliferation in LLC-PK1 cells. **A** LLC-PK1 cells were pretreated with CQ for 2 h followed by mock infection or PDCoV infection (MOI = 2). After adsorption for 2 h, the cells were further cultured in the presence or absence of CQ. Twenty-four hours after infection, the supernatants were harvested and the cell lysates were examined by Western blotting. The relative protein levels of PDCoV-N and the viral titers in the supernatants in **A** are shown in **B** and **C**. **D** LLC-PK1 cells were transfected with siNC and siATG5 for 24 h followed by infected with PDCoV (MOI = 2). Twenty-four hours after infection, the supernatants were harvested and the cell lysates were examined by Western blotting. The relative protein levels of PDCoV-N and the viral titers in the supernatants in **A** are shown in **E** and **F**. **G**–**L** Inhibition of ferroptosis decreases PDCoV replication in LLC-PK1 cells. **G**–**J** LLC-PK1 cells were pretreated with Lip-1 (10 µM) or Fer-1 (50 µM) for 2 h followed by mock infection or PDCoV infection (MOI = 2). After adsorption for 2 h, the cells were further cultured in the presence or absence of Lip-1 or Fer-1. Twenty-four hours after infection, the supernatants were harvested and the cell lysates were examined by Western blotting. The relative protein levels of PDCoV-N in **G** and **J** are shown in **H** and **K**. The viral titers in the supernatants in **G** and **J** are shown in **I** and **L**. Data are representative of three independent experiments and presented as mean ± standard deviation (SD). ***p < 0.001; **p < 0.01; *p < 0.05. **M** Schematic diagram of PDCoV infection-induced autophagy and ferroptosis in LLC-PK1 cells.

Our findings indicated that PDCoV infection induces autophagy, which subsequently activates ferroptosis. To investigate the roles of autophagy and ferroptosis in PDCoV replication, we employed CQ to inhibit autophagy in LLC-PK1 cells. The optimal concentration of CQ was first determined using the CCK-8 assay (Additional file 11 A). As a result, CQ treatment significantly decreased the expression of the N protein in a dose-dependent manner (Figures 7A, B and Additional file 11B), and the viral loads were also reduced following CQ treatment, which is consistent with the results from western blotting (Figure 7C). These results indicate that the inhibition of autophagy by CQ suppresses PDCoV replication. Additionally, knockdown of ATG5 markedly attenuated PDCoV-induced autophagy, leading to a reduction in both the content of viral N protein and viral loads in cell cultures (Figures 7D–F). These findings demonstrate that inhibition of autophagy suppresses PDCoV replication in LLC-PK1 cells. Regarding ferroptosis, we selected two specific inhibitors, liproxstatin-1 (Lip-1) and ferrostatin-1 (Fer-1), to pretreat cells and assess the impact of ferroptosis on PDCoV replication. Consequently, both Lip-1 (Figures 7G, H) and Fer-1 (Figures 7J, K) treatment resulted in decreased expression of viral N protein. More notably, the viral loads in cell cultures were significantly reduced in both the Lip-1 (Figure 7I) and Fer-1 (Figure 7L) treated groups compared with controls. To further validate above finding, cells were treated with CQ and Lip-1, and viral titers in the culture supernatant were measured at different time points post-infection (0, 6, 12, 18, and 24 hpi). The results revealed that, at the early stage of infection (6 hpi), only treatment with the autophagy inhibitor CQ significantly reduced viral titers compared with the virus-alone group, while the ferroptosis inhibitor Lip-1 showed no significant effect. As infection progressed, both inhibitors significantly suppressed viral titers in the culture supernatant in a time-dependent manner (Additional file 12). Collectively, these results demonstrate that both autophagy and ferroptosis are essential for PDCoV infection in LLC-PK1 cells (Figure 7M).

## Discussion

PDCoV is an emerging enteropathogenic coronavirus that causes severe diarrhea in neonatal piglets worldwide and poses a potential risk for cross-species transmission, presenting significant economic losses and public health concern [35]. Exploration of host responses to PDCoV infection is an essential strategy to improve solutions for PDCoV infections [36–38]. Recently, integrated transcriptome and proteome analysis has fully utilized the advantages of these two research areas, enabling a comprehensive exploration of the mechanisms underlying virus–host interactions [39, 40].

In this study, we observed a significant increase in the expression of several innate immunity-related genes/proteins, such as RSAD2, IFITs, ISG15, and OASs. Among them, RSAD2, an important antiviral factor, has functions as an interferon effector protein [41]. Previous studies have demonstrated that RSAD2 was significantly upregulated in IPEC cells after PDCoV infection [42], which is consistent with our findings. The IFITs family (IFIT1, IFIT2, and IFIT3) have been shown to play a significant role in the antiviral response of host cells [43]. It was demonstrated that IFIT1 binds to 2′-O′-methylated RNA, leading to the inhibition of translation and a subsequent decrease in viral infection [44]. These findings suggest that IFITs may serve as potential targets for restricting PDCoV infection. Moreover, we found that FTL, GCLM, SLC25A5, and TUBB4A were downregulated in both the transcriptomic and the proteomic data, which may seriously damage the iron homeostasis, antioxidant defense, energy supply, and the structural integrity of host cells. It was confirmed that Severe acute respiratory syndrome coronavirus 2 (SARS-CoV-2) inhibits the Nrf2 pathway through its multiple proteins, which lead to downregulation of downstream gene expression including antioxidant genes, GCLM (involved in glutathione synthesis), and FTL (involved in iron storage), resulting in compromised cellular antioxidant capacity and iron buffering ability [45, 46]. Therefore, PDCoV infection may also regulate the iron homeostasis through FTL, GCLM, etc. Notably, the regulation of our screened genes in PDCoV-infected cells remains unclear but is critical to understand how PDCoV disrupts host cell redox balance and iron homeostasis to achieve efficient replication and pathogenesis.

GO and KEGG analyses indicated that innate immunity, necroptosis, autophagy, and phagosome were enriched. In PPI analysis, the setting of different thresholds enables an exploration of data reliability and information completeness [47]. A high threshold yields a small and highly reliable network with a low false positive rate, while a low threshold greatly expands the scale of the network, incorporating more predicted, coexpressed, or weak interaction relationships, which can reveal new potential associations but carries a higher risk of false positives [48, 49]. Ont he basis of our experimental dataset, the PPI of the transcriptomic and proteomics set a different score within a reasonable range to construct the network. The results revealed the presence of several autophagy- and ferroptosis-related genes in the center of the network, such as ATG14, ATG9A, ATG4C, and GPX4. Our results can be used as a foundation for future experimental verification. Autophagy is a defense mechanism that maintains cellular homeostasis against external stimuli. However, a variety of viruses hijack cellular autophagy to support their replication [23]. Previous research has demonstrated that TGEV infection can induce mitochondrial autophagy (mitophagy) in IPEC cells mediated by DJ-1 (a multifunctional redox-sensitive protein), which enhances viral replication [49]. PEDV induces autophagy to promote viral replication via protein kinase RNA-like endoplasmic reticulum kinase (PERK) and IRE1 pathways in Vero cells [50]. PDCoV-induced autophagy enhances virus replication via the p38 signaling pathway during PDCoV infection [28]. We found that PDCoV induced autophagy in LLC-PK1, and that inhibition of autophagy decreased viral proliferation. These results indicate that autophagy may be one of the important mechanisms by which coronaviruses promote their own replication.

Regarding ferroptosis, it was reported that Erastin may inhibit the replication of PEDV in Vero cells through the regulation of ferroptosis [51]. Moreover, researchers found that RSL3 inhibits PEDV replication by activating ferroptosis [26]. In the case of PDCoV, it was reported that PDCoV infection induced ferroptosis in IPEC-J2 cells, and the activation of ferroptosis by Erastin significant inhibits the viral replication [18]. These results are inconsistent with our findings, which can be attributed to differences in the genomes of Coronaviridae virus and their respective host cells. In addition, regarding how the activation of autophagy and ferroptosis facilitate PDCoV replication, we speculate that, on one hand, coronaviruses may hijack autophagic initiation to induce membrane rearrangement and form double-membrane vesicles, thereby providing sites for viral replication. On the other hand, the activation of ferroptosis may promote lipid synthesis [52], which in turn facilitates viral envelope formation. Alternatively, the induction of ferroptosis might facilitate viral release. Moreover, it is worth noting that the activation of ferroptosis signaling is closely linked to mitochondrial damage. Given that mitochondria are central organelles in initiating innate immune responses, ferroptosis activation may also, to some extent, attenuate the antiviral effects of innate immunity, thereby favoring viral proliferation.

Ferroptosis has been considered a type of autophagy-dependent cell death due to the selective autophagic degradation of anti-ferroptosis proteins [53]. It was reported that knockdown of Atg5 and Atg7 suppressed Erastin-induced ferroptosis with decreased intracellular ferrous iron levels and lipid peroxidation [54]. In this study, we found that PDCoV infection induced autophagy and ferroptosis, and that inhibition of autophagy suppressed PDCoV-induced ferroptosis, which significantly decreased viral proliferation. Therefore, these results suggest the important role of autophagy in the induction of ferroptosis. Given the important roles of autophagy and ferroptosis in PDCoV replication, further investigation of the intrinsic molecular mechanisms will be expected to develop key targets against PDCoV infection. Notably, although the highly consistent results from both inhibitors support the conclusion that inhibiting ferroptosis suppresses PDCoV replication, we cannot completely rule out potential off-target effects of the inhibitors on related pathways, such as ROS homeostasis or lipid metabolism. Future studies utilizing more specific genetic knockdown approaches or further metabolomic profiling will help to provide more additional data support for this conclusion.

The transcriptomic and proteomic profiles of PDCoV-infected LLC-PK1 cells were obtained at different time points in this study. Through integrated omics analysis, we identified DEGs and DEPs primarily enriched in innate immunity, autophagy, and ferroptosis signaling pathways. Furthermore, we confirmed that the inhibition of autophagy suppresses PDCoV-induced ferroptosis, which further decreases PDCoV replication in LLC-PK1 cells. This research provides new insights into the interactions between PDCoV and hosts and serves as a new theoretical basis for the prevention and control of PDCoV infection.

## Supplementary Information

**Additional file 1.** **Characterization of PDCoV infection in LLC-PK1 cells.** (A) The CPE of PDCoV-infected LLC-PK cells at 1.5 h post-infection and 18 h post-infection. MOI=2. Scale Bar, 100 μm. (B) IFA was conducted to assess the virus infection status. Cells were fixed, and virus-infected cells were visualized through immunofluorescence (IF) staining of the PDCoV N protein (green). Cell nuclei were stained with DAPI (blue). Scale bar, 100 μm.

**Additional file 2.** **Sample sequencing data quality in PDCoV-infected LLC-PK1 cells.** Table representing the Raw reads, Clean reads, the percentage of bases with a Phred value greater than 20 and 30 among the total bases and the percentages of G and C among the four bases in clean reads.

**Additional file 3.** **Quality evaluation and transcriptomic enrichment analysis of the DEGs.** (A) The boxplot distribution of gene expression level. (B) GO annotations of the all DEGs at 1.5 h post-infection. The x-axis represents the GO function and the y-axis was the -log_10_ (padj) value. (C) GO annotations of the all DEGs at 18 h post-infection. The x-axis represent she GO function and the y-axis was the -log_10_ (padj) value. (D, E) Top 20 of enriched KEGG for up-regulated and down-regulated DEGs at 1.5 h post-infection. (F, G) Top 20 of enriched KEGG for up-regulated and down-regulated DEGs at 18 h post-infection. (H, I) PPI of up-regulated and down-regulated DEGs at 18 h post-infection. The size of the circles and the depth of the colors indicated the numbers of interacted proteins and the Fold Change values.

**Additional file 4. Top 10 upregulated and downregulated DEGs following LLC-PK1 cells at 1.5 h post-PDCoV infection.** Table representing the top 10 upregulated and downregulated DEGs at 1.5 h post-PDCoV infection, sorted based on the log₂FoldChange value.

**Additional file 5. Top 10 upregulated and downregulated DEGs following LLC-PK1 cells at 18 h post-PDCoV infection.** Table representing the top 10 upregulated and downregulated DEGs at 18 h post-PDCoV infection, sorted based on the log₂FoldChange value.

**Additional file 6. Top 50 transcriptomics KEGG in LLC-PK1 cells at 18 post-PDCoV infection.** Table representing the top 50 of transcriptomics KEGG and their ID, Description, *P* value, Gene Ratio, and partial Gene Name.

**Additional file 7. Top 5 genes in four subclusters on LLC-PK1 cells.** The table of the top 5 genes, based on log2(fpkm+1) values, includes the gene ID, name, and description.

**Additional file 8.** **Proteomics quality evaluation of the identified proteins in PDCoV-infected LLC-PK1 cells at 18 h post-infection.** (A) PCA analysis based on the protein expression profile of each sample. (B) Repeatability CV analysis for all proteins. (C) Peptide length distributions of all identified proteins. (D) The near-zero distribution of mass error of all identified proteins. (E) Unique peptide segment distribution of all identified proteins. (F, G) The identified proteins coverage and molecular weight distribution. (H) Functional categories of the identified proteins were annotated by the COGs database. (I-K) TF, KEGG pathway, IPR annotations of the identified proteins.

**Additional file 9.** **Top 20 DEPs in LLC-PK1 cells at 18 post-PDCoV infection. Table representing the top 20 DEPs at 18 h post-PDCoV infection, sorted based on the log₂FoldChange value.**

**Additional file 10.**. **Proteomics enrichment analysis of DEPs in PDCoV-infected LLC-PK1 cells at 18 h post-infection.** (A, B) GO annotations of DEPs including BP, CC, and MF. Top 20 of enriched GO terms for up-regulated (A) and down-regulated DEPs (B) were showed. The x-axis represents the GO terms and the y-axis represents the number and percent of proteins. (C, D) Top 20 of enriched KEGG for up-regulated and down-regulated DEPs. The x-axis represents the GeneRatio and the y-axis was the KEGG description. The color of -log_10_ (*p* value) coded from blue (low) to red (high). Number is the number of DEPs annotated to the KEGG pathway.

**Additional file 11.** **The effect of CQ at different concentrations on the viability of LLC-PK1 cells. **(A) LLC-PK1 cells were treated with CQ at different concentrations for 24 h. Subsequently, the effect of CQ on cell viability was assessed using a CCK-8 assay kit. (B) LLC-PK1 cells were pretreated with CQ (2, 10, and 50µM) for 2 h followed by mock infection or PDCoV infection (MOI = 2). After adsorption for 2 h, the cells were further cultured in the presence or absence of CQ. After 24 hours of PDCoV infection, the cells lysates were harvested and examined by western blotting.

**Additional file 12.** **Viral titers across different experimental groups (Virus alone, Virus with autophagy inhibitor, Virus with ferroptosis inhibitor) at multiple time points (0, 6, 12, 18, and 24 hpi).** LLC-PK1 cells were pretreated with Lip-1 (10 µM) or CQ (50 µM) for 2 h followed by mock infection or PDCoV infection (MOI = 2). After adsorption for 2 h, the cells were further cultured in the presence or absence of Lip-1 or CQ for different time (0, 6, 12, 18 and 24 h). And then, the viral titers in the supernatants were detected by TCID_50_ assay. Data are representative of three independent experiments and presented as means ± standard deviation (SD). ***, *p* < 0.001; **, *p* < 0.01; *, *p* < 0.05.

## Data Availability

The PDCoV sequences obtained in this study have been submitted to GenBank under accession no. PQ373831.2. All data generated during this study are included in this published article and its Supplementary Information files. All the data for the statistical analysis, datasets required to replicate all study findings reported in the article, and raw data for images have been deposited in the Science Data Bank repository [55]. All raw data of transcriptome have been uploaded to the Gene Expression Omnibus (GEO) of the National Center for Biotechnology Information (NCBI; accession no. GSE302490). All proteome sequencing data have been deposited to the ProteomeXchange Consortium [56] via the iProX partner repository with dataset identifier PXD066341.

## References

[1] Yao X, Lu WH, Qiao WT, Zhang YQ, Zhang BY, Li HX, Li JL (2025) The highly pathogenic strain of porcine deltacoronavirus disrupts the intestinal barrier and causes diarrhea in newborn piglets. Virulence 16:2446742 39758030 10.1080/21505594.2024.2446742 PMC12915422

[2] Shan X, Li R, Ma X, Qiu G, Xiang Y, Zhang X, Wu D, Wang L, Zhang J, Wang T, Li W, Xiang Y, Song H, Niu D (2024) Epidemiology, pathogenesis, immune evasion mechanism and vaccine development of porcine deltacoronavirus. Funct Integr Genomics 24:79 38653845 10.1007/s10142-024-01346-7

[3] Jin XH, Zhang YF, Yuan YX, Han L, Zhang GP, Hu H (2021) Isolation, characterization and transcriptome analysis of porcine deltacoronavirus strain HNZK-02 from Henan Province, China. Mol Immunol 134:86–99 33740580 10.1016/j.molimm.2021.03.006

[4] Dong N, Fang LR, Zeng SL, Sun QQ, Chen HC, Xiao SB (2015) Porcine deltacoronavirus in mainland China. Emerg Infect Dis 21:2254–2255 26584185 10.3201/eid2112.150283 PMC4672429

[5] Wang LY, Byrum B, Zhang Y (2014) Detection and genetic characterization of deltacoronavirus in pigs, Ohio, USA, 2014. Emerg Infect Dis 20:1227–1230 24964136 10.3201/eid2007.140296 PMC4073853

[6] Thakor JC, Dinesh M, Manikandan R, Bindu S, Sahoo M, Sahoo D, Dhawan M, Pandey MK, Tiwari R, Emran TB, Dhama K, Chaicumpa W (2022) Swine coronaviruses (SCoVs) and their emerging threats to swine population, inter-species transmission, exploring the susceptibility of pigs for SARS-CoV-2 and zoonotic concerns. Vet Q 42:125–147 35584308 10.1080/01652176.2022.2079756 PMC9225692

[7] Woo PC, Lau SK, Lam CS, Lau CC, Tsang AK, Lau JH, Bai R, Teng JL, Tsang CC, Wang M, Zheng BJ, Chan KH, Yuen KY (2012) Discovery of seven novel mammalian and avian coronaviruses in the genus deltacoronavirus supports bat coronaviruses as the gene source of alphacoronavirus and betacoronavirus and avian coronaviruses as the gene source of gammacoronavirus and deltacoronavirus. J Virol 86:3995–4008 22278237 10.1128/JVI.06540-11 PMC3302495

[8] Li WT, Hulswit RJG, Kenney SP, Widjaja L, Jung K, Alhamo MA, Dieren BV, Kuppeveld FJMV, Saif LJ, Bosch BJ (2018) Broad receptor engagement of an emerging global coronavirus may potentiate its diverse cross-species transmissibility. Proc Natl Acad Sci USA 115:135–143 10.1073/pnas.1802879115 PMC5984533 29760102

[9] Lednicky JA, Tagliamonte MS, White SK, Elbadry MA, Alam MM, Stephenson CJ, Bonny TS (2021) Independent infections of porcine deltacoronavirus among Haitian children. Nature 600:133–137 34789872 10.1038/s41586-021-04111-z PMC8636265

[10] Bahoussi AN, Wang PH, Shah PT, Bu HL, Wu CX, Li X (2022) Evolutionary plasticity of zoonotic porcine deltacoronavirus (PDCoV): genetic characteristics and geographic distribution. Vet Res 18:444 10.1186/s12917-022-03554-4 PMC9772601 36550483

[11] Li MW, Guo LJ, Li F (2022) Interplay between swine enteric coronaviruses and host innate immune. Front Vet Sci 9:1083605 36619958 10.3389/fvets.2022.1083605 PMC9814124

[12] Chen J, Fang PX, Wang MH, Peng Q, Ren J, Wang D, Peng GQ, Fang LR, Xiao SB, Ding Z (2019) Porcine deltacoronavirus nucleocapsid protein antagonizes IFN β production by impairing dsRNA and PACT binding to RIG I. Virus Genes 55:520–531 31129785 10.1007/s11262-019-01673-z PMC7088841

[13] Fang PX, Fang LR, Xia SJ, Ren J, Zhang JS, Bai DC, Zhou YR, Peng GQ, Zhao SH, Xiao SB (2020) Porcine deltacoronavirus accessory protein NS7a antagonizes IFN-β production by competing with TRAF3 and IRF3 for binding to IKKε. Front Cell Infect Microbiol 10:257 32656094 10.3389/fcimb.2020.00257 PMC7326017

[14] Chen YM, Burrough E (2022) The effects of Swine Coronaviruses on ER stress, autophagy, apoptosis, and alterations in cell morphology. Pathogens 11:940 36015060 10.3390/pathogens11080940 PMC9416022

[15] Duan C, Wang JC, Liu Y, Zhang JR, Si JY, Hao ZH, Wang JF (2021) Antiviral effects of ergosterol peroxide in a pig model of porcine deltacoronavirus (PDCoV) infection involves modulation of apoptosis and tight junction in the small intestine. Vet Res 52:86 34127062 10.1186/s13567-021-00955-5 PMC8201433

[16] Tiano SML, Landi N, Marano V, Ragucci S, Bianco G, Cacchiarelli D, Swuec P, Silva M, De Cegli R, Sacco F, Di Maro A, Cortese M (2024) Quinoin, type 1 ribosome inactivating protein alters SARS-CoV-2 viral replication organelle restricting viral replication and spread. Int J Biol Macromol 280:135700 39288862 10.1016/j.ijbiomac.2024.135700

[17] Bi XJ, Liu W, Ding X, Liang S, Zheng YF, Zhu XL, Quan S, Yi X, Xiang N, Du JP (2022) Proteomic and metabolomic profiling of urine uncovers immune responses in patients with COVID-19. Cell Rep 38:110271 35026155 10.1016/j.celrep.2021.110271 PMC8712267

[18] Wang GZ, Cao YA, Xu C, Zhang SS, Huang YJ, Zhang S, Bao WB (2024) Comprehensive transcriptomic and metabolomic analysis of porcine intestinal epithelial cells after PDCoV infection. Front Vet Sci 11:1359547 38855411 10.3389/fvets.2024.1359547 PMC11160942

[19] Zhao MZ, Li L, Zhai LH, Yue Q, Liu HY, Ren SP, Jiang XW, Gao FH, Bai SS, Li HH (2020) Comparative transcriptomic and proteomic analyses prove that IFN-λ1 is a more potent inducer of ISGs than IFN-α against porcine epidemic diarrhea virus in porcine intestinal epithelial cells. J Proteome Res 19:3697–3707 32692179 10.1021/acs.jproteome.0c00164

[20] Jin S, He X, Ma L, Zhuang Z, Wang Y, Lin M, Cai S, Wei L, Wang Z, Zhao Z, Wu Y, Sun L, Li C, Xie W, Zhao Y, Songyang Z, Peng K, Zhao J, Cui J (2022) Suppression of ACE2 SUMOylation protects against SARS-CoV-2 infection through TOLLIP-mediated selective autophagy. Nat Commun 13:204 10.1038/s41467-022-32957-y PMC9440653 36057605

[21] Xie YC, Kang R, Klionsky DJ, Tang DL (2023) GPX4 in cell death, autophagy, and disease. Autophagy 19:2621–2638 37272058 10.1080/15548627.2023.2218764 PMC10472888

[22] Yuan FE, Sun Q, Zhang S, Ye LG, Xu Y, Deng G, Xu Z, Zhang SQ, Liu BH, Chen QX (2022) The dual role of p62 in ferroptosis of glioblastoma according to p53 status. Cell Biosci 12:20 35216629 10.1186/s13578-022-00764-z PMC8881833

[23] Chen L, Wei MZ, Zhou BJ, Wang KG, Zhu EP, Cheng ZT (2024) The roles and mechanisms of endoplasmic reticulum stress-mediated autophagy in animal viral infections. Vet Res 55:107 39227990 10.1186/s13567-024-01360-4 PMC11373180

[24] Zhang B, Li LY, Wang N, Zhu ZX, Wang MY, Tan WP, Liu JF, Zhou SH (2025) A new pathway for ferroptosis regulation: The PRMTs. Int J Biol Macromol 285:138143 39622375 10.1016/j.ijbiomac.2024.138143

[25] Zhu Q, Liu T, Qin W, Yang X, Tong W, Yu H, Zheng H, Tong G, Shan T, Zhang Y, Liu X, Kong N (2025) BTG3 inhibits porcine epidemic diarrhea virus replication by promoting viral S2 protein degradation through the autophagy and proteasome pathways. Vet Microbiol 302:110402 39842367 10.1016/j.vetmic.2025.110402

[26] Li YG, Bao YW, Li Y, Duan XX, Dong SM, Lin JX, Chang XY, Tan Y, Zhang HL, Shan H (2023) RSL3 inhibits Porcine Epidemic Diarrhea Virus replication by activating ferroptosis. Viruses 15:2080 37896857 10.3390/v15102080 PMC10612067

[27] Qin P, Du EZ, Luo WT, Yang YL, Zhang YQ, Wang B, Huang YW (2019) Characteristics of the life cycle of porcine deltacoronavirus (PDCoV) in vitro: replication kinetics, cellular ultrastructure and virion morphology, and evidence of inducing autophagy. Viruses 11:455 31109068 10.3390/v11050455 PMC6563515

[28] Duan C, Liu Y, Hao ZH, Wang JF (2021) Ergosterol peroxide suppresses porcine deltacoronavirus (PDCoV)-induced autophagy to inhibit virus replication via p38 signaling pathway. Vet Microbiol 257:109068 33894664 10.1016/j.vetmic.2021.109068 PMC8035807

[29] Yang X, Yin H, Liu M, Wang X, Song T, Song A, Xi Y, Zhang T, Sun Z, Li W, Niu S, Zainab F, Wang C, Zhang D, Wang H, Yang B (2025) Isolation, phylogenetics, and characterization of a new PDCoV strain that affects cellular gene expression in human cells. Front Microbiol 16:1534907 40207165 10.3389/fmicb.2025.1534907 PMC11979167

[30] Wu T, Hu E, Xu S, Chen M, Guo P, Dai Z, Feng T, Zhou L, Tang W, Zhan L, Fu X, Liu S, Bo X, Yu G (2021) ClusterProfiler 4.0: a universal enrichment tool for interpreting omics data. Innovation 2:100141 34557778 10.1016/j.xinn.2021.100141 PMC8454663

[31] Cell-PLoc 2.0: a package of web-servers for predicting subcellular localization of proteins in different organisms. http://www.csbio.sjtu.edu.cn/bioinf/Cell-PLoc-2/. 10.1038/nprot.2007.494 18274516

[32] Gene Set Enrichment Analysis. http://software.broadinstitute.org/gsea/index.jsp.

[33] STRING: protein-protein interaction networks functional enrichment analysis. https://cn.string-db.org/.

[34] Bader GD, Hogue CWV (2003) An automated method for finding molecular complexes in large protein interaction networks. BMC Bioinformatics 4:2 12525261 10.1186/1471-2105-4-2 PMC149346

[35] Du WJ, Debski-Antoniak O, Drabek D, Haperen RV, Dortmondt MV, Lee JVD, Drulyte L, Kuppeveld FJMV, Grosveld F, Hurdiss DL, Bosch BJ (2024) Neutralizing antibodies reveal cryptic vulnerabilities and interdomain crosstalk in the porcine deltacoronavirus spike protein. Nat Commun 15:5330 38909062 10.1038/s41467-024-49693-0 PMC11193727

[36] Jin XH, Wu XY, Li ZH, Hu YX, Xia L, Zu SP, Zhang GP, Hu H (2024) Integrin αVβ3 mediates porcine deltacoronavirus infection and inflammatory response through activation of the FAK-PI3K-AKT-nf-κB signalling pathway. Virulence 15:2407847 39368071 10.1080/21505594.2024.2407847 PMC11457627

[37] Zhou XR, Ge XN, Zhang YN, Han J, Guo X, Chen YH, Zhou L, Yang HC (2021) Attenuation of porcine deltacoronavirus disease severity by porcine reproductive and respiratory syndrome virus coinfection in a weaning pig model. Virulence 12:1011–1021 33797313 10.1080/21505594.2021.1908742 PMC8023240

[38] Zhang S, Cao YA, Huang YJ, Zhang XL, Mou CX, Qin T, Chen ZH, Bao WB (2025) Abortive PDCoV infection triggers Wnt/β-catenin pathway activation, enhancing intestinal stem cell self-renewal and promoting chicken resistance. J Virol 98:00137–00225 10.1128/jvi.00137-25 PMC11998530 40135895

[39] Zhang H, Yu QS, Guo R, Zhu Q, Yu J, Zhang ZH, Lan L, Tang C, Yu CQ, Zhang B (2025) Integrative transcriptomic and proteomic analyses reveal that carbon metabolism and complement system of Madin Darby Bovine Kidney cells are affected by bovine coronavirus infection. Vet Res 21:398 10.1186/s12917-025-04848-z PMC12131655 40457360

[40] Ma L, Lin X, Xu M, Ke XL, Liu D, Chen QJ (2025) Exploring the biological mechanisms of severe COVID-19 in the elderly: insights from an aged mouse model. Virulence 16:2487671 40228062 10.1080/21505594.2025.2487671 PMC12005417

[41] Zhen YN, Ding BB (2024) Coronavirus hijacks STX18-ATG14 axis-regulated lipophagy to evade an anti-viral effect. Autophagy 20:1895–1896 38477940 10.1080/15548627.2024.2330039 PMC11262221

[42] Cruz-Pulido D, Boley PA, Ouma WZ, Saif LJ, Kenney SP (2021) Comparative transcriptome profiling of human and pig intestinal epithelial cells after Porcine Deltacoronavirus infection. Viruses 13:292 33668405 10.3390/v13020292 PMC7918119

[43] Huang HX, Lei XX, Zhao CC, Qin Y, Li YY, Zhang XY, Li CK, Lan T, Zhao BP, Sun WC, Lu HJ, Jin NY (2024) Porcine deltacoronavirus nsp5 antagonizes type I interferon signaling by cleaving IFIT3. J Virol 98:1–19 10.1128/jvi.01682-23 PMC10878044 38289117

[44] Yao JL, Yang Z, Guo XC, Wang JC, Yu BL, Liu SG, Hu XM, Yang KK, Yao LG, Zhang T (2024) Recombinant porcine interferon δ8 inhibited porcine deltacoronavirus infection in vitro and in vivo. Int J Biol Macromol 279:135375 39244115 10.1016/j.ijbiomac.2024.135375

[45] Ramezani A, Nahad MP, Faghihloo E (2018) The role of Nrf2 transcription factor in viral infection. J Cell Biochem 119:6366–6382 29737559 10.1002/jcb.26897

[46] Dodson M, Castro-Portuguez R, Zhang DD (2019) Nrf2 plays a critical role in mitigating lipid peroxidation and ferroptosis. Redox Biol 23:101107 30692038 10.1016/j.redox.2019.101107 PMC6859567

[47] Liu X, Maiorino E, Halu A, Glass K, Prasad RB, Loscalzo J, Gao JX, Sharma A (2020) Robustness and lethality in multilayer biological molecular networks. Nat Commun 11:6043 33247151 10.1038/s41467-020-19841-3 PMC7699651

[48] Jeong H, Mason S, Barabási AL, Oltvai Z (2001) Lethality and centrality in protein networks. Nature 411:41 11333967 10.1038/35075138

[49] Zhu L, Mou CX, Yang X, Lin J, Yang Q (2016) Mitophagy in TGEV infection counteracts oxidative stress and apoptosis. Oncotarget 7:27122–27141 27027356 10.18632/oncotarget.8345 PMC5053637

[50] Sun P, Jin J, Wang LX, Zhou HC, Zhang Q, Xu XG (2021) Porcine epidemic diarrhea virus infections induce autophagy in Vero cells via ROS-dependent endoplasmic reticulum stress through PERK and IRE1 pathways. Vet Microbiol 253:108959 33360915 10.1016/j.vetmic.2020.108959

[51] Zhang HL, Li YG, Yang RM, Xiao L, Dong SM, Lin JX, Liu G, Shan H (2023) Erastin inhibits porcine epidemic diarrhea virus replication in Vero cells. Front Cell Infect Microbiol 13:1142173 36936772 10.3389/fcimb.2023.1142173 PMC10015705

[52] Dong W, Du K, Ding Y, Liu Y, Peng L, Wu C, Sun Y, Li Z, Niu Y (2024) FAdV-4-induced ferroptosis affects fat metabolism in LMH cells. Vet Microbiol 293:110068 38579482 10.1016/j.vetmic.2024.110068

[53] Hou W, Xie YC, Song XX, Sun XF, Lotxe MT, Zeh HJ 3^rd^, Kang R, Tang DL (2016) Autophagy promotes ferroptosis by degradation of ferritin. Autophagy 12:1425–1428 27245739 10.1080/15548627.2016.1187366 PMC4968231

[54] Bai YS, Meng LJ, Han L, Jia YY, Zhao YN, Gao H, Kang R, Wang XF, Tang DL, Dai EY (2019) Lipid storage and lipophagy regulates ferroptosis. Biochem Biophys Res Commun 508:997–1003 30545638 10.1016/j.bbrc.2018.12.039

[55] Science Data Bank repository. 10.57760/sciencedb.27821.

[56] ProteomeXchange Consortium. https://proteomecentral.proteomexchange.org.

