# Supplementary figures and images for: Integrated analysis of transcriptome and proteome reveal that PDCoV infection induces autophagy-dependent ferroptosis to facilitate viral replication
Source: Vet Res. 2026 May 18;57:77. doi: 10.1186/s13567-026-01724-y (PMC13181929; doi:10.1186/s13567-026-01724-y)

**A**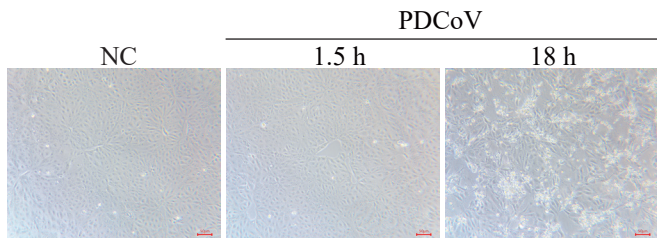**B**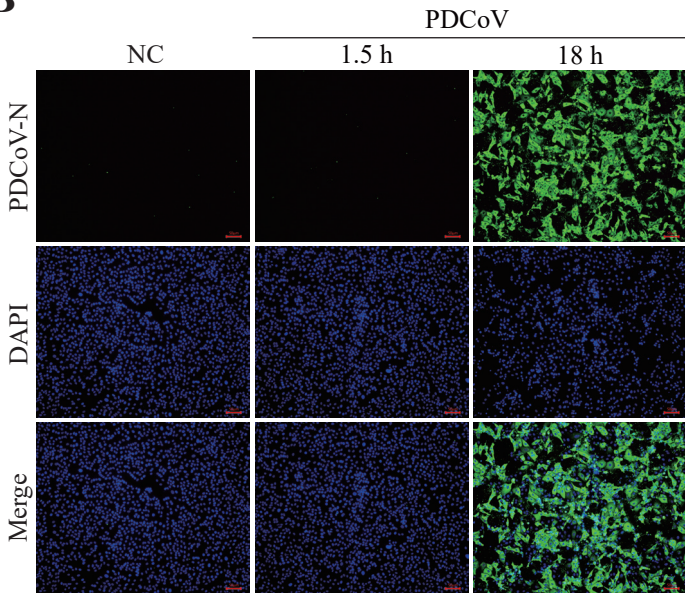

Supplement: Supplementary file 1 — Additional file 1. Characterization of PDCoV infection in LLC-PK1 cells. (A) The CPE of PDCoV-infected LLC-PK cells at 1.5 h post-infection and 18 h post-infection. MOI=2. Scale Bar, 100 μm. (B) IFA was conducted to assess the virus infection status. Cells were fixed, and virus-infected cells were visualized through immunofluorescence (IF) staining of the PDCoV N protein (green). Cell nuclei were stained with DAPI (blue). Scale bar, 100 μm. [file 13567_2026_1724_MOESM1_ESM.pdf]

A

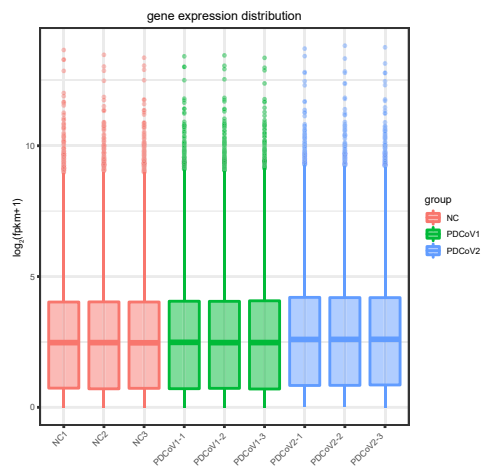

B

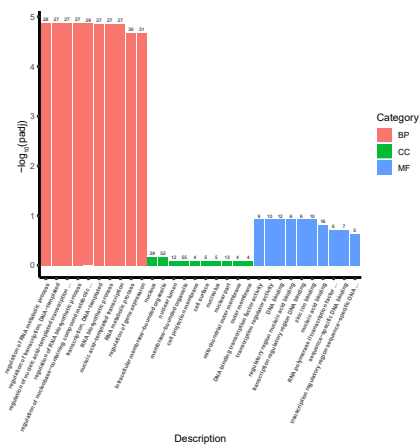

C

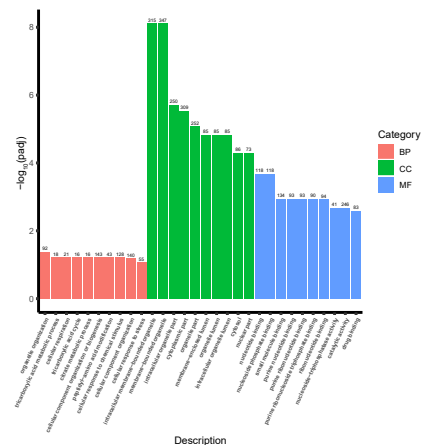

D

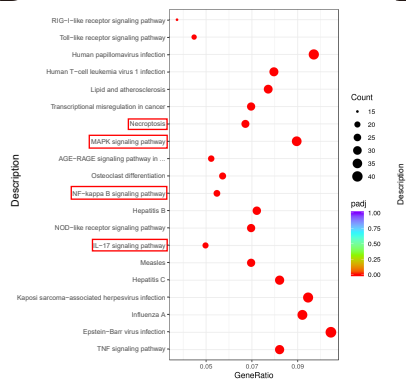

E

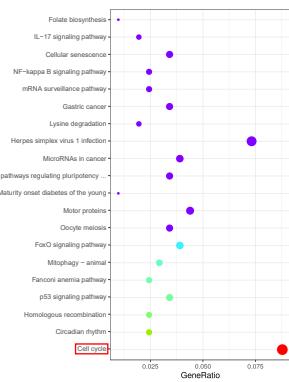

F

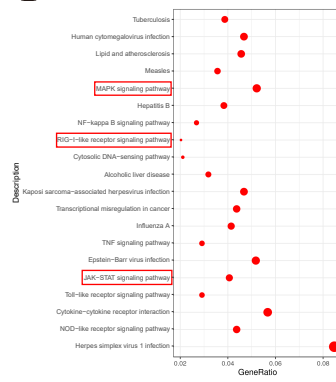

G

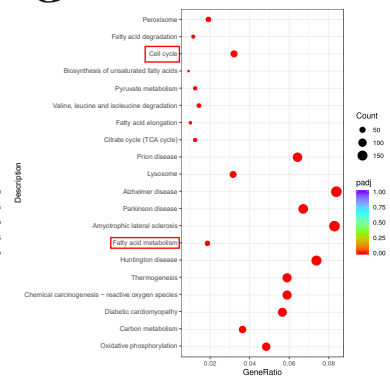

H

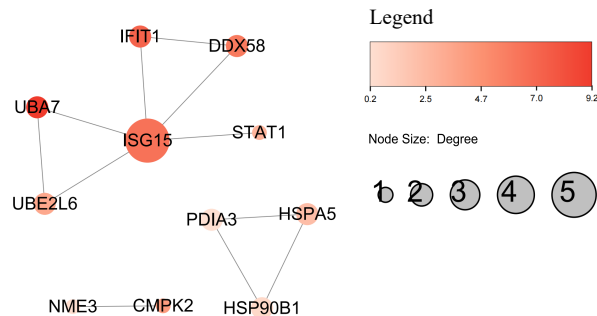

I

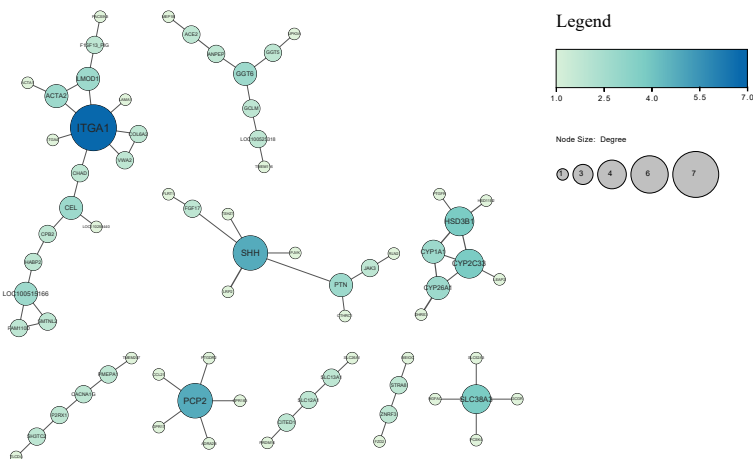

Supplement: Supplementary file 3 — Additional file 3. Quality evaluation and transcriptomic enrichment analysis of the DEGs. (A) The boxplot distribution of gene expression level. (B) GO annotations of the all DEGs at 1.5 h post-infection. The x-axis represents the GO function and the y-axis was the -log10 (padj) value. (C) GO annotations of the all DEGs at 18 h post-infection. The x-axis represent she GO function and the y-axis was the -log10 (padj) value. (D, E) Top 20 of enriched KEGG for up-regulated and down-regulated DEGs at 1.5 h post-infection. (F, G) Top 20 of enriched KEGG for up-regulated and down-regulated DEGs at 18 h post-infection. (H, I) PPI of up-regulated and down-regulated DEGs at 18 h post-infection. The size of the circles and the depth of the colors indicated the numbers of interacted proteins and the Fold Change values. [file 13567_2026_1724_MOESM3_ESM.pdf]

A

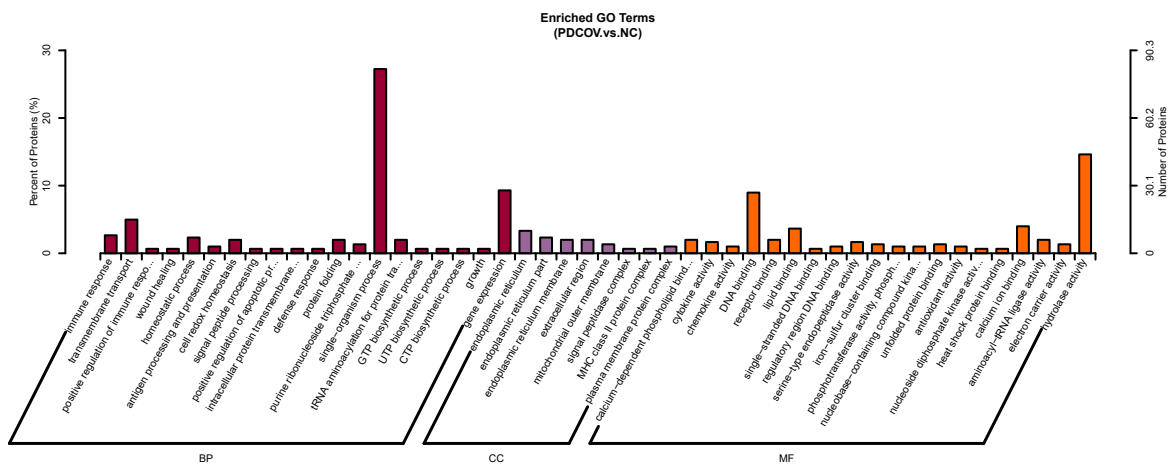

B

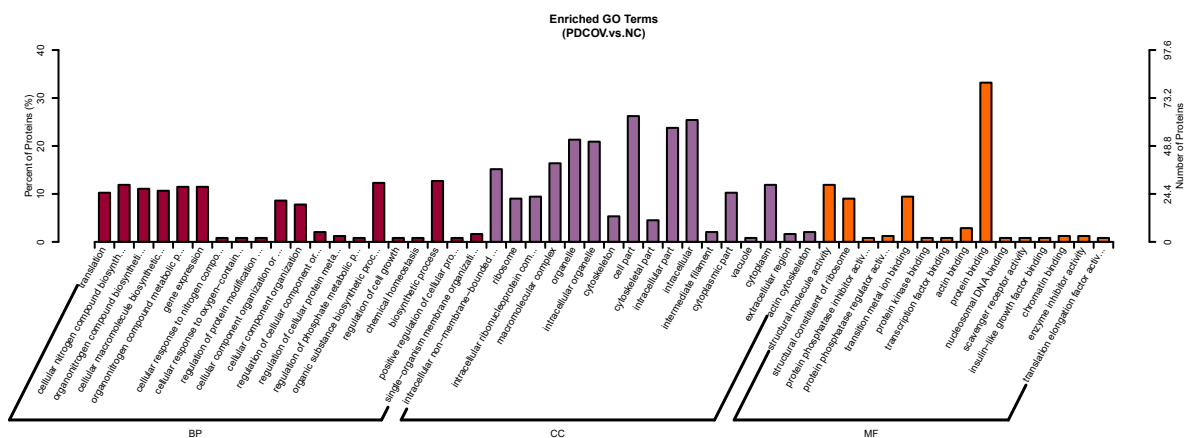

C

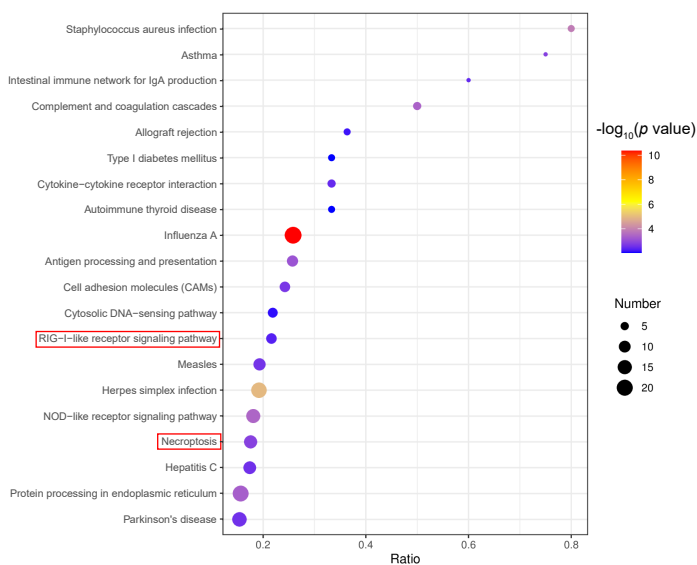

D

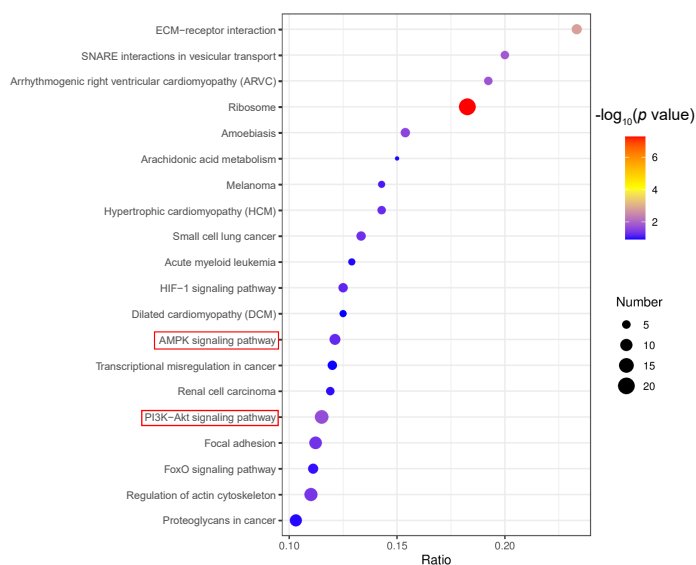

Supplement: Supplementary file 10 — Additional file 10.. Proteomics enrichment analysis of DEPs in PDCoV-infected LLC-PK1 cells at 18 h post-infection. (A, B) GO annotations of DEPs including BP, CC, and MF. Top 20 of enriched GO terms for up-regulated (A) and down-regulated DEPs (B) were showed. The x-axis represents the GO terms and the y-axis represents the number and percent of proteins. (C, D) Top 20 of enriched KEGG for up-regulated and down-regulated DEPs. The x-axis represents the GeneRatio and the y-axis was the KEGG description. The color of -log10 (p value) coded from blue (low) to red (high). Number is the number of DEPs annotated to the KEGG pathway. [file 13567_2026_1724_MOESM10_ESM.pdf]

**A**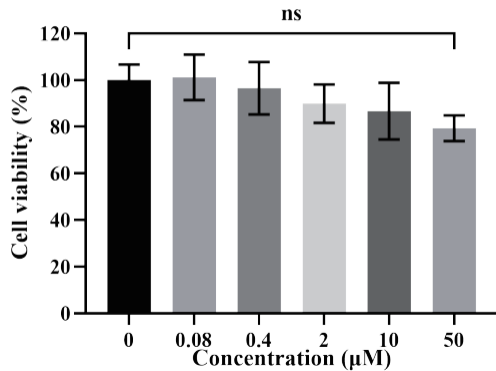**B**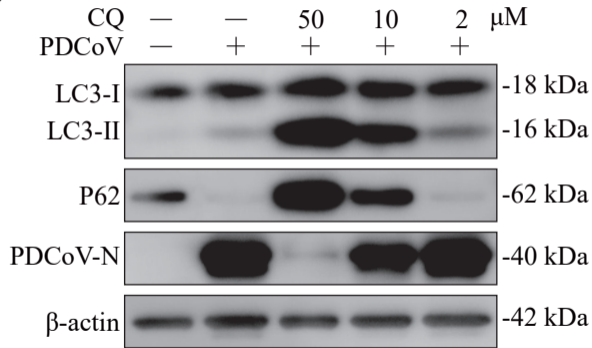

Supplement: Supplementary file 11 — Additional file 11. The effect of CQ at different concentrations on the viability of LLC-PK1 cells. (A) LLC-PK1 cells were treated with CQ at different concentrations for 24 h. Subsequently, the effect of CQ on cell viability was assessed using a CCK-8 assay kit. (B) LLC-PK1 cells were pretreated with CQ (2, 10, and 50µM) for 2 h followed by mock infection or PDCoV infection (MOI = 2). After adsorption for 2 h, the cells were further cultured in the presence or absence of CQ. After 24 hours of PDCoV infection, the cells lysates were harvested and examined by western blotting. [file 13567_2026_1724_MOESM11_ESM.pdf]

**A**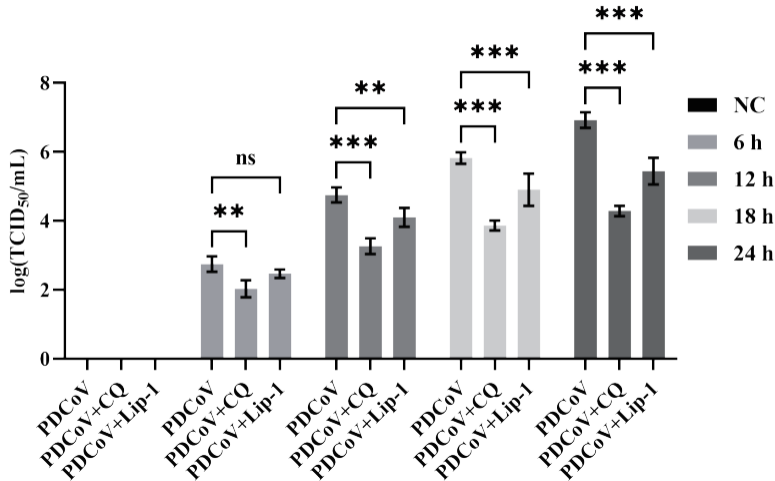

Supplement: Supplementary file 12 — Additional file 12. Viral titers across different experimental groups (Virus alone, Virus with autophagy inhibitor, Virus with ferroptosis inhibitor) at multiple time points (0, 6, 12, 18, and 24 hpi). LLC-PK1 cells were pretreated with Lip-1 (10 µM) or CQ (50 µM) for 2 h followed by mock infection or PDCoV infection (MOI = 2). After adsorption for 2 h, the cells were further cultured in the presence or absence of Lip-1 or CQ for different time (0, 6, 12, 18 and 24 h). And then, the viral titers in the supernatants were detected by TCID50 assay. Data are representative of three independent experiments and presented as means ± standard deviation (SD). ***, p < 0.001; **, p < 0.01; *, p < 0.05. [file 13567_2026_1724_MOESM12_ESM.pdf]
