# Supplementary material for: Integrated analysis of transcriptome and proteome reveal that PDCoV infection induces autophagy-dependent ferroptosis to facilitate viral replication
Source: Vet Res. 2026 May 18;57:77. doi: 10.1186/s13567-026-01724-y (PMC13181929; doi:10.1186/s13567-026-01724-y)
Supplement: Supplementary file 2 — Additional file 2. Sample sequencing data quality in PDCoV-infected LLC-PK1 cells. Table representing the Raw reads, Clean reads, the percentage of bases with a Phred value greater than 20 and 30 among the total bases and the percentages of G and C among the four bases in clean reads. [file 13567_2026_1724_MOESM2_ESM.pdf]

**Sample sequencing data quality in PDCoV-infected LLC-PK1 cells**

| Sample | Raw Reads | Raw Bases | Clean Reads | Clean Bases | Q20 | Q30 | GC Pct |
|---|---|---|---|---|---|---|---|
| NC1 | 43106964 | 6.47G | 41408792 | 6.21G | 98.10 | 94.64 | 49.81 |
| NC2 | 62324340 | 9.35G | 57330820 | 8.6G | 97.97 | 94.29 | 51.34 |
| NC3 | 55860204 | 8.38G | 53575754 | 8.04G | 97.95 | 94.30 | 51.40 |
| PDCoV1-1 | 43682852 | 6.55G | 42811160 | 6.42G | 97.93 | 94.23 | 51.47 |
| PDCoV1-2 | 50134992 | 7.52G | 48873148 | 7.33G | 98.02 | 94.45 | 51.40 |
| PDCoV1-3 | 43042630 | 6.46G | 41914392 | 6.29G | 98.03 | 94.47 | 52.07 |
| PDCoV2-1 | 80174200 | 12.03G | 78587752 | 11.79G | 98.63 | 96.01 | 49.34 |
| PDCoV2-2 | 125523786 | 18.83G | 123557262 | 18.53G | 98.59 | 95.91 | 48.84 |
| PDCoV2-3 | 126402764 | 18.96G | 123931108 | 18.59G | 98.66 | 96.14 | 49.00 |

Q20: The percentage of bases with a Phred value greater than 20 among the total bases

Q30: The percentage of bases with a Phred value greater than 30 among the total bases

GC pct: The percentages of G and C among the four bases in clean reads

PDCoV1: LLC-PK1 cells at 1.5 h post-PDCoV infection

PDCoV2: LLC-PK1 cells at 18 h post-PDCoV infection
