# Supplementary material for: Integrated analysis of transcriptome and proteome reveal that PDCoV infection induces autophagy-dependent ferroptosis to facilitate viral replication
Source: Vet Res. 2026 May 18;57:77. doi: 10.1186/s13567-026-01724-y (PMC13181929; doi:10.1186/s13567-026-01724-y)
Supplement: Supplementary file 4 — Additional file 4. Top 10 upregulated and downregulated DEGs following LLC-PK1 cells at 1.5 h post-PDCoV infection. Table representing the top 10 upregulated and downregulated DEGs at 1.5 h post-PDCoV infection, sorted based on the log₂FoldChange value. [file 13567_2026_1724_MOESM4_ESM.pdf]

Top 10 upregulated and downregulated DEGs following LLC-PK1 cells at 1.5 h post-PDCoV infection

Gene ID	log_2Fold Change	Gene name	Gene description
100515875	2.75	BATF2	basic leucine zipper ATF-like transcription factor 2
396752	2.15	RSAD2	radical S-adenosyl methionine domain containing 2
100622968	2.15	DUSP6	dual specificity phosphatase 6
595128	1.98	OAS2	2'-5'-oligoadenylate synthetase 2
100523358	1.94	JUNB	JUNB proto-oncogene
100520726	1.90	EGR1	early growth response 1
100623872	1.51	CMPK2	cytidine/uridine monophosphate kinase 2
100738544	1.46	ERRFI1	ERBB receptor feedback inhibitor 1
396611	1.33	IRF1	interferon regulatory factor 1
100514873	1.29	BHLHE40	basic helix-loop-helix family member e40
100286778	-1.83	PDK4	pyruvate dehydrogenase kinase 4
100626099	-0.81	TBL2	transducin beta like 2
102163816	-0.67	LOC102163816	uncharacterized LOC102163816
100522864	-0.64	RNPC3	RNA binding region (RNP1%2C RRM) containing 3
100525703	-0.57	DNAJB4	DNAJ heat shock protein family (Hsp40) member B4
100513133	-0.50	LOC100513133	uncharacterized LOC100513133
100520233	-0.47	ARRDC3	arrestin domain containing 3
100155928	-0.46	LRIG3	leucine rich repeats and immunoglobulin like domains 3
100623242	-0.41	FEM1C	fem-1 homolog C
100521330	-0.32	SKIDA1	SKI/DACH domain containing 1
