# Supplementary material for: Integrated analysis of transcriptome and proteome reveal that PDCoV infection induces autophagy-dependent ferroptosis to facilitate viral replication
Source: Vet Res. 2026 May 18;57:77. doi: 10.1186/s13567-026-01724-y (PMC13181929; doi:10.1186/s13567-026-01724-y)
Supplement: Supplementary file 5 — Additional file 5. Top 10 upregulated and downregulated DEGs following LLC-PK1 cells at 18 h post-PDCoV infection. Table representing the top 10 upregulated and downregulated DEGs at 18 h post-PDCoV infection, sorted based on the log₂FoldChange value. [file 13567_2026_1724_MOESM5_ESM.pdf]

Top 10 upregulated and downregulated DEGs following LLC-PK1 cells at 18 h post-PDCoV infection

Gene ID	log_2Fold Change	Gene name	Gene description
494019	10.89	CXCL10	C-X-C motif chemokine ligand 10
100169744	10.57	CXCL11	C-X-C motif chemokine ligand 11
396668	8.68	CCL4	C-C motif chemokine ligand 4
110255360	8.18	LOC110255360	interferon-induced very large GTPase 1-like
100155467	8.00	IFIT2	interferon induced protein with tetratricopeptide repeats 2
100154248	7.71	IFIT3	interferon induced protein with tetratricopeptide repeats 3
100155195	7.69	LOC100155195	guanylate-binding protein 7
595119	7.68	OASL	2'-5'-oligoadenylate synthetase like
397623	7.57	SIGLEC1	sialic acid binding Ig like lectin 1
100154284	7.46	PRDM1	PR/SET domain 1%2C transcript variant X8
397520	-4.30	ANPEP	alanyl aminopeptidase
102161049	-3.72	FAT2	FAT atypical cadherin 2
100124374	-3.30	CYP26A1	cytochrome P450%2C family 26
100519689	-3.18	LRP2	LDL receptor related protein 2
100152510	-3.13	CTHRC1	collagen triple helix repeat containing 1
100151826	-2.83	VAV3	vav guanine nucleotide exchange factor 3
100515029	-2.28	ADGRG1	adhesion G protein-coupled receptor G1
100627510	-1.99	CDH6	cadherin 6
397403	-1.99	GPX1	glutathione peroxidase 1
100624725	-1.56	NPNT	nephronectin
