# Supplementary material for: Integrated analysis of transcriptome and proteome reveal that PDCoV infection induces autophagy-dependent ferroptosis to facilitate viral replication
Source: Vet Res. 2026 May 18;57:77. doi: 10.1186/s13567-026-01724-y (PMC13181929; doi:10.1186/s13567-026-01724-y)
Supplement: Supplementary file 6 — Additional file 6. Top 50 transcriptomics KEGG in LLC-PK1 cells at 18 post-PDCoV infection. Table representing the top 50 of transcriptomics KEGG and their ID, Description, P value, Gene Ratio, and partial Gene Name. [file 13567_2026_1724_MOESM6_ESM.pdf]

Top 50 transcriptomics KEGG in LLC-PK1 cells at 18 post-PDCoV infection

KEGG ID	Description	*P* value	Gene Ratio	Gene Name (partial)
ssc04621	NOD-like receptor signaling pathway	1.19E-11	142/4696	OAS2/STAT1/TXNIP/TNFAIP3/GBP1/BIRC3/STAT2/OAS1/IRF7/NFKB1
ssc04620	Toll-like receptor signaling pathway	5.55E-08	86/4696	CXCL10/STAT1/CXCL11/IRF7/NFKB1/CCL5/NFKBIA/CD40/IL6/CCL4
ssc04068	FoxO signaling pathway	9.31E-08	104/4696	GADD45A/TNFSF10/IL6/TGFB3/PIK3R1/BCL6/GADD45G/PLK3/IKBKB/STAT3
ssc05165	Human papillomavirus infection	2.71E-07	234/4696	ISG15/MX1/STAT1/IRF1/STAT2/TCIRG1/HES4/NFKB1/OASL/IFNAR2
ssc04668	TNF signaling pathway	3.42E-07	89/4696	CSF1/CXCL10/TNFAIP3/BIRC3/ICAM1/IRF1/JUNB/NFKB1/CCL5/NFKBIA
ssc04144	Endocytosis	5.65E-07	178/4696	PML/SLA7/HSP70.2/EHD4/CHMP7/EHD3/LDLR/ARAP2/HSPA6/SLA-1
ssc04010	MAPK signaling pathway	1.31E-06	195/4696	CSF1/DUSP5/MYC/GADD45A/DUSP1/NFKB1/DUSP6/DUSP10/TGFB3/MAP3K8
ssc05208	Chemical carcinogenesis - reactive oxygen species	2.39E-06	194/4696	NFKB1/NFKBIA/ABL2/PIK3R1/FOS/JUN/MAP3K14/PTPN1/PRKD2/ABL1
ssc05220	Chronic myeloid leukemia	2.59E-06	64/4696	MYC/GADD45A/NFKB1/NFKBIA/TGFB3/PIK3R1/E2F2/GADD45G/STAT5A/ABL1
ssc04919	Thyroid hormone signaling pathway	2.76E-06	94/4696	STAT1/MYC/PIK3R1/RCAN1/MED13/BMP4/ATP1B1/PDPK1/PLCB3/DIO
ssc05161	Hepatitis B	5.49E-06	134/4696	DDX58/STAT1/MYC/STAT2/IRF7/NFKB1/IFIH1/NFKBIA/CASP10/IL6
ssc04622	RIG-I-like receptor signaling pathway	5.62E-06	62/4696	DDX58/ISG15/CXCL10/ADAR/DHX58/IRF7/NFKB1/IFIH1/NFKBIA/CASP1
ssc05418	Fluid shear stress and atherosclerosis	7.09E-06	115/4696	ICAM1/DUSP1/NFKB1/EDN1/VCAM1/IL1A/SDC4/PIK3R1/ARHGEF2/FOS
ssc05203	Viral carcinogenesis	8.82E-06	145/4696	IRF7/NFKB1/NFKBIA/PMAIP1/SLA7/EIF2AK2/PIK3R1/NFKB2/IRF9/ATF
ssc04210	Apoptosis	1.13E-05	115/4696	MCL1/BIRC3/GADD45A/LOC100624226/NFKB1/NFKBIA/TNFSF10/CFLAR/CASP10/LOC100737977
ssc05206	MicroRNAs in cancer	1.19E-05	123/4696	MCL1/TIMP3/MYC/PLAU/DDIT4/NFKB1/RASSF1/PIK3R1/E2F2/SLC7A1
ssc04936	Alcoholic liver disease	1.66E-05	102/4696	NFKB1/NFKBIA/IL6/CXCL2/C2/TICAM1/MAP3K14/C3/RIPK1/C4A

ssc05169	Epstein-Barr virus infection	1.99E-05	168/4696	OAS2/DDX58/ISG15/CXCL10/STAT1/TNFAIP3/TAP1/ICAM1/MYC/GADD45A
ssc05202	Transcriptional misregulation in cancer	2.15E-05	143/4696	BIRC3/MYC/GADD45A/PLAU/PML/NFKB1/NUPR1/CD40/DUSP6/IL6
ssc04630	JAK-STAT signaling pathway	2.80E-05	123/4696	MCL1/STAT1/MYC/STAT2/IL6/JAK2/IFNAR2/PIK3R1/IRF9/LIF
ssc01212	Fatty acid metabolism	3.85E-05	47/4696	CPT1A/ACADSB/ACSL3/HADHA/ACOX1/ACAT2/ACSL5/ACSBG1/OXSM/PPT2
ssc04623	Cytosolic DNA-sensing pathway	4.62E-05	62/4696	DDX58/CXCL10/ADAR/IRF7/NFKB1/CCL5/NFKBIA/ZBP1/IL6/CCL4
ssc04722	Neurotrophin signaling pathway	4.76E-05	95/4696	NFKB1/NFKBIA/NFKBIE/IRAK2/PIK3R1/ATF4/JUN/RIPK2/ABL1/MAP3K5
ssc05167	Kaposi sarcoma-associated herpesvirus infection	4.77E-05	155/4696	STAT1/ICAM1/MYC/STAT2/IRF7/NFKB1/NFKBIA/ZFP36/IL6/CXCL2
ssc05132	Salmonella infection	5.78E-05	187/4696	BIRC3/MYC/LOC100624226/NFKB1/NFKBIA/TNFSF10/LOC100522887/IL6/LOC100737977/NLRP3
ssc05417	Lipid and atherosclerosis	9.26E-05	164/4696	HSPA5/ICAM1/LOC100624226/IRF7/NFKB1/CCL5/NFKBIA/TNFSF10/XBP1/CD40
ssc05219	Bladder cancer	0.000105	34/4696	MYC/RASSF1/E2F2/HBEGF/VEGFA/CDH1/CXCL8/CDKN1A/ERBB2/MDM2
ssc05160	Hepatitis C	0.000105	124/4696	RSAD2/OAS2/DDX58/CXCL10/MX1/STAT1/MYC/STAT2/OAS1/IRF7
ssc04142	Lysosome	0.000107	97/4696	TCIRG1/GM2A/CTSS/NCOA7/AP1S3/ENTPD4/LITAF/M6PR/SCARB2/IDS
ssc05164	Influenza A	0.000124	131/4696	RSAD2/OAS2/DDX58/CXCL10/MX1/STAT1/ADAR/ICAM1/LOC100624226/STAT2
ssc04110	Cell cycle	0.000161	116/4696	MYC/GADD45A/TGFB3/E2F2/ORC2/GADD45G/CDC25B/CDKN2C/ABL1/CDC25A
ssc04072	Phospholipase D signaling pathway	0.000162	105/4696	PLPP3/GNA13/PIK3R1/PTK2B/RALGDS/RRAS2/ADCY4/PLCB3/CYTH1/CXCL8
ssc05133	Pertussis	0.00018	58/4696	IRF1/NFKB1/IL6/C1R/C1S/SERPING1/IL1A/C2/NLRP3/FOS

ssc05163	Human cytomegalovirus infection	0.000227	167/4696	TAP1/MYC/NFKB1/CCL5/NFKBIA/IL6/CCL4/SLA-7/BID/GNA13
ssc04625	C-type lectin receptor signaling pathway	0.000242	76/4696	STAT1/IRF1/STAT2/NFKB1/NFKBIA/IL6/PIK3R1/NFKB2/IRF9/NLRP3
ssc04071	Sphingolipid signaling pathway	0.000292	90/4696	NFKB1/SGPP2/SGMS2/BID/GNA13/PIK3R1/MAP3K5/TNF/TRAF2/PDPK1
ssc05152	Tuberculosis	0.000342	134/4696	STAT1/TCIRG1/NFKB1/CASP10/IL6/JAK2/CD74/TGFB3/IL1A/IRAK2
ssc05010	Alzheimer disease	0.000382	289/4696	CSF1/NFKB1/XBP1/IL6/DDIT3/IL1A/EIF2AK2/BID/PIK3R1/ATF4
ssc05166	Human T-cell leukemia virus 1 infection	0.000633	166/4696	EGR1/ICAM1/MYC/NFKB1/NFKBIA/CD40/ZFP36/IL6/FOSL1/TGFB3
ssc05212	Pancreatic cancer	0.000691	59/4696	STAT1/GADD45A/NFKB1/TGFB3/PIK3R1/E2F2/RALGDS/GADD45G/VEGFA/IKBKB
ssc04145	Phagosome	0.000816	109/4696	TAP1/TCIRG1/C1R/SLA-7/SLADRA/CTSS/OLR1/TAP2/C3/SLA-1
ssc05205	Proteoglycans in cancer	0.000817	143/4696	TIMP3/MYC/PLAU/SDC4/PIK3R1/VAV3/RRAS2/FLNC/PLAUR/HBEGF
ssc04218	Cellular senescence	0.000909	116/4696	MYC/ZFP36L1/GADD45A/NFKB1/IL6/TGFB3/IL1A/SLA-7/PIK3R1/E2F2
ssc04141	Protein processing in endoplasmic reticulum	0.001009	117/4696	HSPA5/HERPUD1/XBP1/DDIT3/HSP70.2/EIF2AK2/ATF4/PPP1R15A/DNAJB1/YOD1
ssc05168	Herpes simplex virus 1 infection	0.00107	252/4696	OAS2/DDX58/STAT1/TAP1/BIRC3/STAT2/OAS1/PML/IRF7/NFKB1
ssc00280	Valine, leucine and isoleucine degradation	0.001105	40/4696	IL4I1/AACS/ACADSB/BCAT1/ALDH2/HADHA/ACAT2/ALDH3A2/DLD/MUT
ssc01200	Carbon metabolism	0.001105	94/4696	HK2/CAT/PDHB/PHGDH/PSAT1/ACOX1/IDH1/ACAT2/GPT2/ME1
ssc04140	Autophagy - animal	0.001133	103/4696	DDIT4/CFLAR/PIK3R1/ERN1/RRAS2/PDPK1/PRKCD/ATG9A/IGF1R/RUBCN
ssc04933	AGE-RAGE signaling pathway in diabetic complications	0.001145	72/4696	EGR1/STAT1/ICAM1/NFKB1/IL6/JAK2/EDN1/TGFB3/VCAM1/IL1A

ssc05216	Thyroid cancer	0.001335	30/4696	MYC/GADD45A/GADD45G/CDH1/CCDC6/BAK1/CDKN1A/TCF7L2/LEF1/DDB2
