# Supplementary material for: Integrated analysis of transcriptome and proteome reveal that PDCoV infection induces autophagy-dependent ferroptosis to facilitate viral replication
Source: Vet Res. 2026 May 18;57:77. doi: 10.1186/s13567-026-01724-y (PMC13181929; doi:10.1186/s13567-026-01724-y)
Supplement: Supplementary file 7 — Additional file 7. Top 5 genes in four subclusters on LLC-PK1 cells. The table of the top 5 genes, based on log2(fpkm+1) values, includes the gene ID, name, and description. [file 13567_2026_1724_MOESM7_ESM.pdf]

Top 5 different genes in four subclusters on LLC-PK1 cells

Gene_id	NC1	NC2	NC3	PDCoV1-1	PDCoV1-2	PDCoV1-3	PDCoV2-1	PDCoV2-2	PDCoV2-3	Gene_name	Gene description
Cluster1											
397422	-2.50	-3.61	-3.20	-2.03	-2.25	-2.31	5.23	5.28	5.39	CCL2	chemokine (C-C motif) ligand 2
396668	-3.50	-2.80	-2.96	-2.12	-2.23	-1.94	5.23	5.17	5.15	CCL4	C-C motif chemokine ligand 4
100310806	-2.58	-2.58	-2.58	-2.58	-2.58	-2.58	5.11	5.27	5.07	IFN-ALPHAOMEGA	interferon-alphaomega
100155467	-2.75	-2.90	-3.03	-2.06	-1.99	-1.89	4.80	4.88	4.93	IFIT2	interferon induced protein with tetratricopeptide repeats 2
100217388	-2.53	-2.68	-2.46	-2.18	-2.32	-2.41	4.90	4.77	4.90	IL29	interleukin 29
Cluster2											
100155760	-1.34	-1.36	-1.10	-1.50	-1.52	-1.05	2.37	2.60	2.90	LOC100155760	interferon omega 1
100522257	-1.44	-1.36	-1.21	-1.32	-1.37	-1.34	2.71	2.52	2.80	IL4I1	interleukin 4 induced 1
100523310	-1.43	-1.27	-1.44	-1.45	-1.13	-1.39	2.68	2.68	2.76	LOC100523310	guanylate-binding protein 6
106504234	-1.00	-1.49	-1.24	-1.03	-1.37	-1.43	2.46	2.38	2.72	LOC106504234	putative olfactory receptor 2I1
100271930	-1.28	-1.26	-1.17	-1.33	-1.33	-1.33	2.51	2.54	2.65	IL7R	interleukin 7 receptor
Cluster3											
397520	1.16	1.15	1.24	1.18	1.09	1.13	-2.36	-2.25	-2.34	ANPEP	alanyl aminopeptidase
100152510	1.14	0.97	1.00	0.86	0.93	0.89	-1.89	-1.81	-2.08	CTHRC1	collagen triple helix repeat containing 1
397134	0.95	1.00	1.02	1.06	1.07	0.91	-2.07	-2.03	-1.93	DAO	D-amino acid oxidase
100516512	1.19	1.10	1.13	0.80	0.85	0.80	-2.10	-1.86	-1.92	FAM107A	family with sequence

											similarity 107 member A
100124374	0.97	1.06	1.02	1.00	0.90	1.04	-2.00	-2.08	-1.90	CYP26A1	cytochrome P450%2C family 26
Cluster4											
494019	-3.34	-3.49	-3.58	-3.19	-2.94	-3.40	6.55	6.66	6.73	CXCL10	C-X-C motif chemokine ligand 10
100169744	-3.16	-3.23	-3.45	-2.66	-3.22	-3.14	6.15	6.33	6.39	CXCL11	C-X-C motif chemokine ligand 11
445459	-2.97	-2.91	-2.63	-3.13	-3.13	-3.13	5.93	6.02	5.94	IFNB1	interferon beta 1
110255217	-2.84	-2.84	-2.84	-2.84	-2.84	-2.73	5.68	5.60	5.65	LOC110255217	interferon lambda-3-like
