# Supplementary material for: Integrated analysis of transcriptome and proteome reveal that PDCoV infection induces autophagy-dependent ferroptosis to facilitate viral replication
Source: Vet Res. 2026 May 18;57:77. doi: 10.1186/s13567-026-01724-y (PMC13181929; doi:10.1186/s13567-026-01724-y)
Supplement: Supplementary file 8 — Additional file 8. Proteomics quality evaluation of the identified proteins in PDCoV-infected LLC-PK1 cells at 18 h post-infection. (A) PCA analysis based on the protein expression profile of each sample. (B) Repeatability CV analysis for all proteins. (C) Peptide length distributions of all identified proteins. (D) The near-zero distribution of mass error of all identified proteins. (E) Unique peptide segment distribution of all identified proteins. (F, G) The identified proteins coverage and molecular weight distribution. (H) Functional categories of the identified proteins were annotated by the COGs database. (I-K) TF, KEGG pathway, IPR annotations of the identified proteins. [file 13567_2026_1724_MOESM8_ESM.pdf]

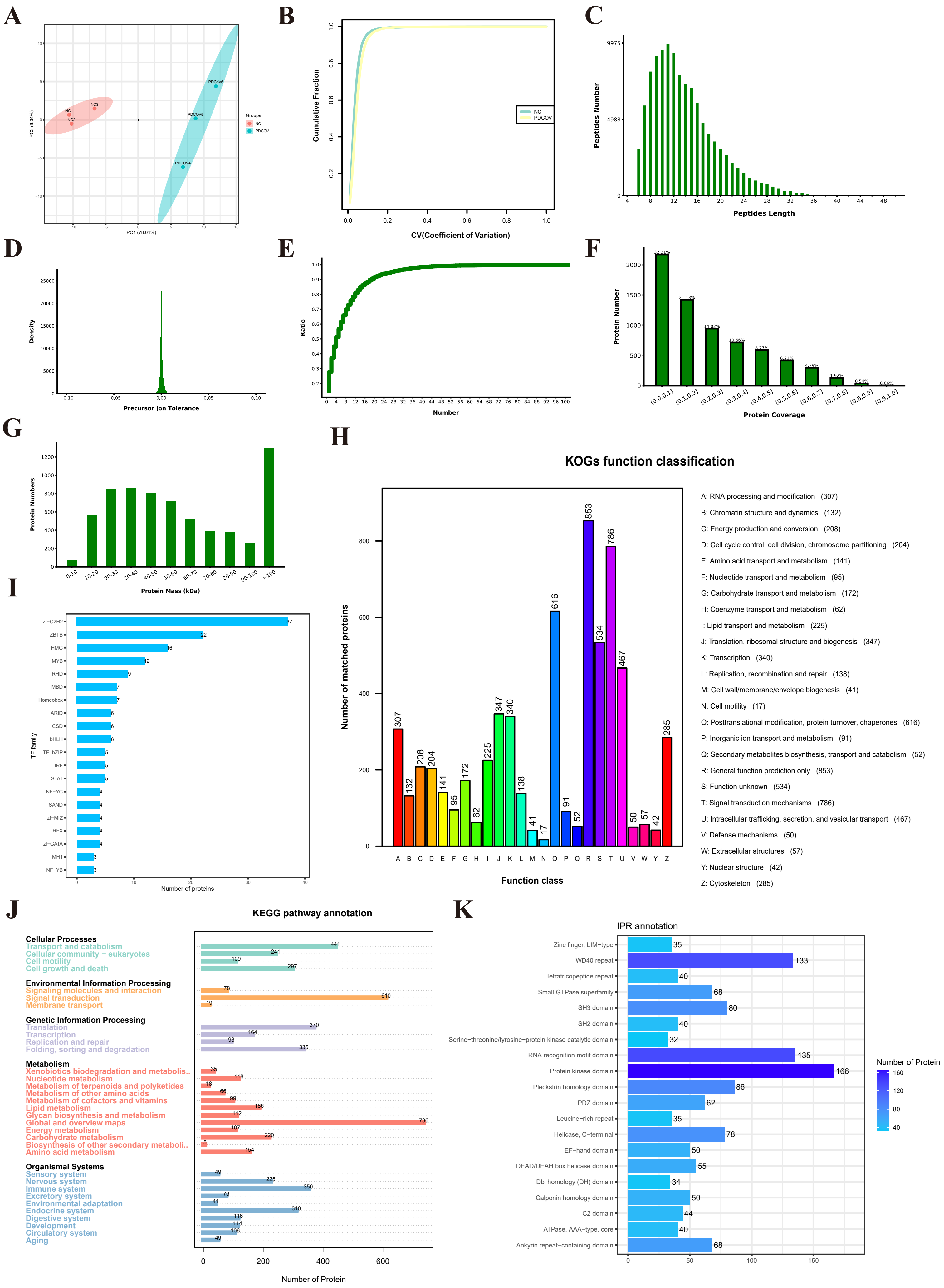

A
PC1 (78.01%)
PC2 (9.04%)
NC1
NC2
NC3
PDCoV4
PDCoV5
PDCoV6
Groups
NC
PDCOV
B
Cumulative Fraction
CV(Coefficient of Variation)
NC
PDCOV
C
Peptides Number
Peptides Length
D
Density
Precursor Ion Tolerance
E
Ratio
Number
F
Protein Number
Protein Coverage
32.31%
21.13%
14.02%
10.66%
8.77%
6.21%
4.39%
1.92%
0.54%
0.06%
G
Protein Numbers
Protein Mass (kDa)
H
KOGs function classification
Number of matched proteins
Function class
A: RNA processing and modification (307)
B: Chromatin structure and dynamics (132)
C: Energy production and conversion (208)
D: Cell cycle control, cell division, chromosome partitioning (204)
E: Amino acid transport and metabolism (141)
F: Nucleotide transport and metabolism (95)
G: Carbohydrate transport and metabolism (172)
H: Coenzyme transport and metabolism (62)
I: Lipid transport and metabolism (225)
J: Translation, ribosomal structure and biogenesis (347)
K: Transcription (340)
L: Replication, recombination and repair (138)
M: Cell wall/membrane/envelope biogenesis (41)
N: Cell motility (17)
O: Posttranslational modification, protein turnover, chaperones (616)
P: Inorganic ion transport and metabolism (91)
Q: Secondary metabolites biosynthesis, transport and catabolism (52)
R: General function prediction only (853)
S: Function unknown (534)
T: Signal transduction mechanisms (786)
U: Intracellular trafficking, secretion, and vesicular transport (467)
V: Defense mechanisms (50)
W: Extracellular structures (57)
Y: Nuclear structure (42)
Z: Cytoskeleton (285)
I
TF family
Number of proteins
zf-C2H2 37
ZBTB 22
HMG 16
MYB 12
RHD 9
MBD 7
Homeobox 7
ARID 6
CSD 6
bHLH 6
TF_bZIP 5
IRF 5
STAT 5
NF-YC 4
SAND 4
zf-MIZ 4
RFX 4
zf-GATA 4
MH1 3
NF-YB 3
J
KEGG pathway annotation
Cellular Processes
Transport and catabolism 441
Cellular community − eukaryotes 241
Cell motility 109
Cell growth and death 297
Environmental Information Processing
Signaling molecules and interaction 78
Signal transduction 610
Membrane transport 19
Genetic Information Processing
Translation 370
Transcription 164
Replication and repair 93
Folding, sorting and degradation 335
Metabolism
Xenobiotics biodegradation and metabolis.. 35
Nucleotide metabolism 118
Metabolism of terpenoids and polyketides 18
Metabolism of other amino acids 66
Metabolism of cofactors and vitamins 99
Lipid metabolism 186
Glycan biosynthesis and metabolism 112
Global and overview maps 736
Energy metabolism 107
Carbohydrate metabolism 220
Biosynthesis of other secondary metaboli.. 5
Amino acid metabolism 154
Organismal Systems
Sensory system 49
Nervous system 225
Immune system 350
Excretory system 76
Environmental adaptation 41
Endocrine system 310
Digestive system 116
Development 114
Circulatory system 106
Aging 49
Number of Protein
K
IPR annotation
Zinc finger, LIM−type 35
WD40 repeat 133
Tetratricopeptide repeat 40
Small GTPase superfamily 68
SH3 domain 80
SH2 domain 40
Serine−threonine/tyrosine−protein kinase catalytic domain 32
RNA recognition motif domain 135
Protein kinase domain 166
Pleckstrin homology domain 86
PDZ domain 62
Leucine−rich repeat 35
Helicase, C−terminal 78
EF−hand domain 50
DEAD/DEAH box helicase domain 55
Dbl homology (DH) domain 34
Calponin homology domain 50
C2 domain 44
ATPase, AAA−type, core 40
Ankyrin repeat−containing domain 68
Number of Protein
