# Supplementary material for: Integrated analysis of transcriptome and proteome reveal that PDCoV infection induces autophagy-dependent ferroptosis to facilitate viral replication
Source: Vet Res. 2026 May 18;57:77. doi: 10.1186/s13567-026-01724-y (PMC13181929; doi:10.1186/s13567-026-01724-y)
Supplement: Supplementary file 9 — Additional file 9. Top 20 DEPs in LLC-PK1 cells at 18 post-PDCoV infection. Table representing the top 20 DEPs at 18 h post-PDCoV infection, sorted based on the log₂FoldChange value. [file 13567_2026_1724_MOESM9_ESM.pdf]

Top 20 DEPs in LLC-PK1 cells at 18 post-PDCoV infection

Protein ID	Gene	*P* value	log_2FC	Up or Down	Description
XP_020953252.1	LOC102165849	0.026298	2.226337	up	uncharacterized protein C1orf167, partial [Sus scrofa]
XP_005671321.1	IFIT2	4.93E-06	2.190216	up	interferon-induced protein with tetratricopeptide repeats 2 isoform X1 [Sus scrofa]
XP_020921771.1	ZCCHC7	5.07E-05	2.124768	up	zinc finger CCHC domain-containing protein 7 [Sus scrofa]
NP_001008691.1	CXCL10	0.001865	2.075217	up	C-X-C motif chemokine 10 precursor [Sus scrofa]
NP_001020387.1	LOC574051	0.002171	1.812561	up	histone H1.2 [Sus scrofa]
XP_003356892.1	LOC100621287	0.012519	1.731126	up	MORF4 family-associated protein 1 [Sus scrofa]
NP_001090885.1	MX2	0.009333	1.730796	up	interferon-induced GTP-binding protein Mx2 [Sus scrofa]
XP_003131510.1	KRT20	0.015711	-1.72269	down	keratin, type I cytoskeletal 20 [Sus scrofa]
XP_020945969.1	LOC100155195	1.95E-07	1.526274	up	guanylate-binding protein 7 [Sus scrofa]
XP_020929339.1	OAS1	4.26E-06	1.448184	up	LOW QUALITY PROTEIN: 2-5-oligoadenylate synthase 1 [Sus scrofa]
NP_001231292.1	IFIT1	0.001463	1.446349	up	interferon-induced protein with tetratricopeptide repeats 1 [Sus scrofa]
NP_998982.1	RSAD2	1.87E-05	1.439903	up	radical S-adenosyl methionine domain-containing protein 2 [Sus scrofa]
XP_020953489.1	LOC110255217	1.55E-05	1.431232	up	interferon lambda-3-like [Sus scrofa]
NP_001191324.1	IFIT3	5.95E-05	1.41343	up	interferon-induced protein with tetratricopeptide repeats 3 [Sus scrofa]
XP_020935903.1	IRF1	9.26E-05	1.374205	up	interferon regulatory factor 1 isoform X1 [Sus scrofa]
NP_999008.2	SOX9	1.08E-05	1.345413	up	transcription factor SOX-9 [Sus scrofa]

XP_020945588.1	LOC100738744	0.000372	-1.30563	down	histone H2B type 2-E [Sus scrofa]
NP_999226.2	MX1	0.006316	1.297035	up	interferon-induced GTP-binding protein Mx1 [Sus scrofa]
XP_005661188.2	SBF2	0.032669	1.283191	up	myotubularin-related protein 13 isoform X1 [Sus scrofa]
XP_020944163.1	GBP1	2.69E-05	1.259154	up	interferon-induced guanylate-binding protein 1 isoform X1 [Sus scrofa]
